# Chain-Length-Optimized 1,8-Naphthalimide–Ibuprofen Fluorescent Probe for HSA Binding and Cellular Imaging

**DOI:** 10.3390/s26216702

**Published:** 2026-10-23

**Authors:** Ding Li, Weichao Ling, Na Liu, Bo Chen, Yang Zhou, Rui Wang, Wei Wei

**Affiliations:** 1 State Key Laboratory of Coordination Chemistry, Chemistry and Biomedicine Innovation Center (ChemBIC), School of Life Sciences, Nanjing University, Nanjing 210023, China; 2 Department of Forensic Medicine, Nanjing Medical University, Nanjing 211166, China; 3 Pingshan Translational Medicine Center, Shenzhen Bay Laboratory, Shenzhen 518055, China; 4 Laboratory of Advanced Theranostic Materials and Technology, Ningbo Institute of Materials Technology and Engineering, Chinese Academy of Sciences, Ningbo 315201, China

**Keywords:** 1,8-naphthalimide, chain-length optimization, fluorescent probe, human serum albumin, cellular imaging

## Abstract

Fluorescent probes based on 1,8-naphthalimide (NI) are attractive molecular tools for sensing and imaging biomacromolecules in biological systems. In this study, a matched series of NI derivatives bearing H, n-hexyl, or n-octyl N-imide substituents and terminal acrylamide or propiolamide groups was designed and synthesized to empirically evaluate how these structural variables affect cell viability and imaging performance. Within this compound series, MTT assays across six cell lines generally yielded higher cell-viability values for the acrylamide-containing probes than for their matched propiolamide analogues, and the octyl-substituted Probe D3 provided a favorable balance between MTT-based cell viability and intracellular fluorescence. Probe D3 exhibited green fluorescence with an emission maximum at approximately 550–560 nm and was compatible with confocal cellular imaging. To introduce HSA-binding capability into the optimized NI scaffold, an ibuprofen-functionalized analogue, Probe D3–ibuprofen, was prepared based on the known binding preference of ibuprofen for Sudlow’s site II of human serum albumin (HSA). HSA caused modest changes in the absorption and fluorescence spectra of Probe D3–ibuprofen, while microscale thermophoresis confirmed a micromolar interaction with an apparent K_d_ value of 14.22 ± 4.43 μM. Quantitative image analysis showed a higher probe-to-DAPI fluorescence intensity ratio in HeLa cells treated with Probe D3–ibuprofen/HSA than in cells treated with the probe alone, supporting its potential for HSA-associated cellular imaging. This work provides a chain-length-guided strategy for engineering NI-based fluorescent probes for protein-oriented cellular imaging.

## 1. Introduction

Fluorescent chemical probes have become indispensable tools in chemical biology and biomedical sensing because they enable the visualization of molecular events with high sensitivity, spatial resolution, and operational simplicity [1,2]. Among the available fluorophore scaffolds, 1,8-naphthalimide (NI) derivatives have attracted sustained attention owing to their strong photostability, chemical robustness, tunable charge-transfer properties, and facile structural modification [3,4,5]. The NI core contains two synthetically addressable regions: the imide nitrogen, which can be used to regulate lipophilicity and cellular permeability, and the aromatic ring, which can be functionalized with electron-donating or recognition motifs to modulate optical output and target responsiveness [6,7]. These structural advantages make NI derivatives particularly suitable for the development of fluorescent sensors, bioimaging probes, and target-responsive molecular reporters. The 1,8-naphthalimide (NI) scaffold used here should be distinguished from naphthalene diimide (NDI) and perylene diimide (PDI), which are chemically distinct bisimide chromophores. Reported NDI- and PDI-based imaging systems frequently rely on controlling aggregation, aqueous dispersibility, and fluorescence switching through donor–acceptor engineering, amphiphilic substitution, or supramolecular assembly [8,9]. In comparison, the present study retains a compact monoimide 4-amino-NI fluorophore. The novelty therefore does not lie in introducing a new fluorophore core, but in systematically comparing matched variations in the N-imide substituent and terminal unsaturated amide before ligand-directed functionalization for HSA-associated imaging. Nevertheless, many NI-based probes still suffer from limited cellular uptake or concentration-dependent cytotoxicity, which restricts their use in biological imaging. Hydrophobic chain engineering is a practical strategy to tune the interaction between small-molecule probes and lipid membranes. Previous studies have shown that hydrocarbon chain length and unsaturation can affect lipid packing, membrane permeability, and transmembrane transport [10]. Therefore, rational optimization of both the N-imide side chain and the electronically active substituent on the NI scaffold is expected to improve the balance among fluorescence performance, cell permeability, and biocompatibility [6,11].

Human serum albumin (HSA) is the most abundant protein in human plasma and plays essential roles in maintaining colloid osmotic pressure and transporting fatty acids, drugs, hormones, and endogenous metabolites [12,13]. Altered HSA concentration, conformation, and ligand-binding capacity are closely associated with liver dysfunction, metabolic disorders, cardiovascular diseases, and inflammatory states [14,15,16,17]. Accordingly, convenient visualization and detection of HSA are important for both fundamental biomedical research and potential diagnostic applications. Compared with conventional analytical methods such as enzyme-linked immunosorbent assay and liquid chromatography-mass spectrometry, fluorescence-based approaches offer simpler operation, higher imaging compatibility, and the possibility of in situ monitoring [18]. For protein-oriented fluorescent probes, the incorporation of a specific ligand can improve target recognition and reduce nonspecific hydrophobic or electrostatic interactions [19]. HSA contains multiple ligand-binding pockets, including the two classical Sudlow binding sites, and ibuprofen is a well-established non-steroidal anti-inflammatory drug with preferential affinity for Sudlow’s site II [20]. This drug-protein recognition pair therefore provides a rational basis for constructing an HSA-responsive NI probe.

**Scheme 1:**
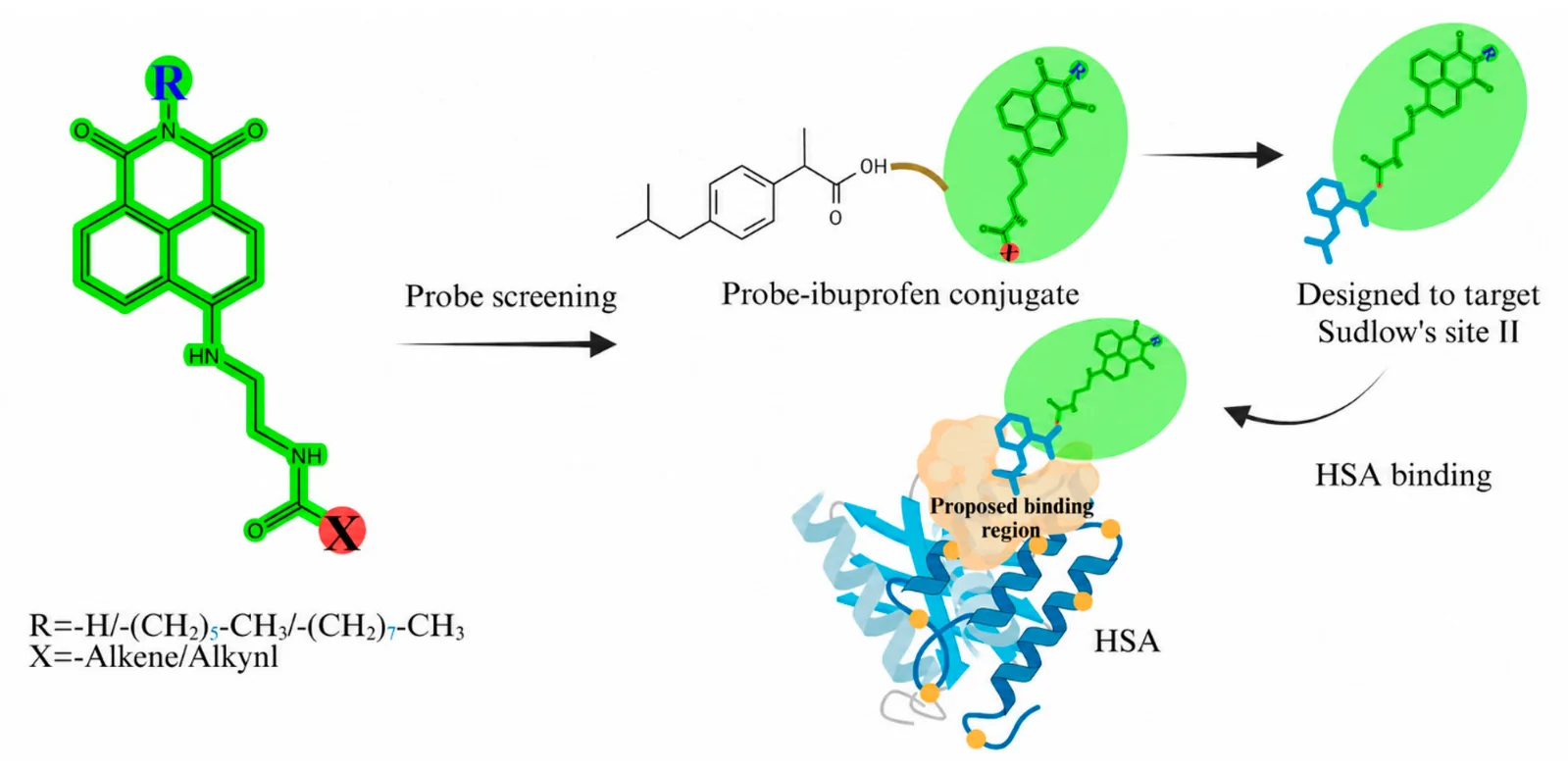
Schematic illustration of the structural optimization of NI-based fluorescent probes and the development of an ibuprofen-functionalized analogue for HSA-associated cellular imaging.

On the basis of this design rationale, six NI-based fluorescent probes were constructed as a matched 2 × 3 molecular series by combining three N-imide substituents (H, n-hexyl, and n-octyl) with either a terminal acrylamide group (D1–D3) or a terminal propiolamide group (E1–E3) (Scheme 1). The N-imide substituents were varied to examine how increasing hydrophobic chain length affects cellular behavior, whereas the matched acrylamide/propiolamide pairs were used to empirically evaluate whether the identity of the terminal unsaturated amide influences cell viability and intracellular fluorescence. These terminal groups were not introduced on the assumption that either an alkene or an alkyne would intrinsically improve cell viability. After cell-based screening, Probe D3, which contains an octyl N-imide substituent and a terminal acrylamide group, was selected because it provided the most favorable balance between MTT-based cell viability and intracellular fluorescence. Probe D3–ibuprofen was subsequently prepared by coupling ibuprofen to the shared octyl-substituted amine intermediate C3 for HSA-associated cellular imaging.

## 2. Materials and Methods

### 2.1. Materials

All chemical reagents and solvents were obtained from commercial suppliers and used without further purification unless otherwise stated. 4-Bromo-1,8-naphthalic anhydride, ethylenediamine, n-hexylamine, n-octylamine, acryloyl chloride, propiolic acid, ibuprofen, 4-dimethylaminopyridine, triethylamine, potassium carbonate, and other reagents used for the synthesis of NI derivatives were purchased from Aladdin (Shanghai, China), Macklin (Shanghai, China), Energy Chemical (Shanghai, China), Sigma-Aldrich (St. Louis, MO, USA), or equivalent suppliers. Organic solvents, including dichloromethane, methanol, ethanol, acetonitrile, dimethyl sulfoxide (DMSO), ethyl acetate, petroleum ether, and N,N-dimethylformamide, were of analytical or chromatographic grade and were obtained from Sinopharm Chemical Reagent Co., Ltd. (Shanghai, China). or other commercial suppliers. Human serum albumin was purchased from Sigma-Aldrich (St. Louis, MO, USA). Phosphate-buffered saline (PBS) was purchased from Solarbio (Beijing, China) or prepared according to standard laboratory protocols.

Dulbecco’s modified Eagle’s medium (DMEM), fetal bovine serum (FBS), penicillin-streptomycin solution, and L-glutamine were obtained from Gibco (Grand Island, NY, USA). MTT, DMSO for cell viability assays, 4′,6-diamidino-2-phenylindole (DAPI), paraformaldehyde, Triton X-100, and Tween-80 were purchased from Sigma-Aldrich (St. Louis, MO, USA), Invitrogen (Carlsbad, CA, USA), Beyotime Biotechnology (Shanghai, China), or equivalent suppliers. Human breast cancer cells (MCF-7), human cervical cancer cells (HeLa), human ovarian cancer cells (A2780), human ovarian teratocarcinoma cells (PA-1), rat pheochromocytoma cells (PC-12), and human embryonic kidney cells (293T) were obtained from ATCC or a certified cell bank. Ultrapure water was prepared using a Milli-Q water purification system. Stock solutions of the synthesized probes were prepared in DMSO and diluted with PBS or culture medium immediately before use. The final DMSO concentration in cell experiments was maintained below 0.1% unless otherwise specified.

### 2.2. Cell Culture

MCF-7, HeLa, A2780, PA-1, PC-12, and 293T cells were cultured in DMEM supplemented with 10% FBS, penicillin-streptomycin, and L-glutamine. Cells were maintained at 37 °C in a humidified incubator containing 5% CO_2_. Cells were passaged at appropriate confluence and used in the logarithmic growth phase for cytotoxicity and imaging experiments.

### 2.3. Cell Viability Assay

Cell viability was evaluated using the MTT colorimetric assay. Briefly, cells were seeded in 96-well plates at a density of 8 × 10^3^ cells per well and allowed to adhere before probe treatment. Probe stock solutions were diluted in complete culture medium to the indicated concentrations (0–150 μM), and cells were incubated with the probes for 24 h. Subsequently, 20 μL of MTT solution (5 mg/mL in PBS) was added to each well, followed by incubation for 4 h at 37 °C. The culture medium was then removed, and 150 μL of DMSO was added to dissolve the formazan crystals. Absorbance was measured at 490 nm using a microplate reader. Cell viability was calculated as the percentage of absorbance relative to untreated control cells.

### 2.4. UV–Visible Absorption and Fluorescence Measurements

Stock solutions of Probe D1–D3 and Probe E1–E3 were prepared in DMSO. For spectroscopic measurements, the stock solutions were diluted to a final probe concentration of 5 μM in PBS containing 1% (*v*/*v*) DMSO and 0.2% (*v*/*v*) Tween 80. UV–visible absorption spectra were recorded over the wavelength range of 350–600 nm. For pH-dependent fluorescence measurements, the pH values of the probe solutions were adjusted to 4.5, 5.5, 6.5, 7.4, and 8.0 while maintaining the same probe concentration and solvent composition. Fluorescence emission spectra were collected from 450 to 800 nm using an excitation wavelength of 440 nm. Fluorescence intensities are reported in arbitrary units. Instrumental settings were kept identical within each experimental comparison. Different acquisition settings were used for different sets of fluorescence measurements; therefore, absolute fluorescence intensities should not be directly compared across datasets acquired under different settings. The photostability of Probe D3 and Probe D3–ibuprofen was assessed using a fluorescence spectrometer. Each probe solution was continuously excited at 440 nm for 10 min (600 s), while its fluorescence emission intensity at 530 nm was continuously monitored.

### 2.5. Fluorescence Imaging Experiments

MCF-7 and HeLa cells were incubated with the corresponding probes at a final concentration of 5 μM for 4 h in complete culture medium. The probe stock solutions were prepared in DMSO and diluted with the culture medium before use, with the final DMSO concentration maintained below 0.1% (*v*/*v*). Cells were fixed with 4% paraformaldehyde in PBS for 15 min and permeabilized with 0.1% Triton X-100 in PBS for 15 min. Nuclei were stained with DAPI before imaging. Fluorescence images were acquired using an LSM 980 laser scanning confocal microscope (Carl Zeiss Microscopy GmbH, Jena, Germany) and processed using ZEN 3.2 blue edition software. The excitation wavelength for NI probes was 445 nm, and emission was collected in the green channel centered at 530 ± 10 nm.

### 2.6. Fluorescence Spectroscopy and HSA-Associated Imaging

The emission spectrum of Probe D3 was recorded in PBS containing 1% (*v*/*v*) DMSO and 0.2% (*v*/*v*) Tween 80 using an excitation wavelength of 440 nm. The UV–visible absorption and fluorescence emission spectra of Probe D3–ibuprofen (5.0 μM) were recorded in the absence and presence of HSA (10.0 μM) under the same solution conditions. Absorption spectra were collected from 300 to 500 nm, and fluorescence emission spectra were collected from 450 to 800 nm using an excitation wavelength of 440 nm. Prepare separate solutions of Probe D3 and Probe D3–ibuprofen at a final concentration of 5.0 μM in PBS (pH 7.4) containing 1% (*v*/*v*) DMSO and 0.2% (*v*/*v*) Tween 80. Add BSA, transferrin (TRF), or IgG individually to each probe solution to obtain a final protein concentration of 10.0 μM. Mix gently and incubate at room temperature (approximately 25 °C) for 3 min. Record fluorescence emission spectra over 450–800 nm using an excitation wavelength of 440 nm on a fluorescence spectrometer. Include probe-only solutions prepared under the same conditions as controls. Keep the excitation and emission slit widths, detector gain, and other acquisition settings identical within each comparison. For HSA-associated cellular imaging, Probe D3-ibuprofen was diluted to the working concentration in culture medium. HeLa cells were incubated with Probe D3-ibuprofen (5 μM) or Probe D3-ibuprofen/HSA complexes (5 μM/10 μM) for 30 min at 37 °C. After incubation, cells were washed with PBS and imaged by confocal microscopy under the indicated excitation and emission settings.

### 2.7. MST Analysis of the Binding Affinity Between Probe D3–Ibuprofen and HSA

The binding affinity between Probe D3–ibuprofen and HSA was determined by microscale thermophoresis (MST). HSA was serially diluted in MST assay buffer and incubated with a fixed concentration of Probe D3–ibuprofen (200 nM). After incubation at room temperature to allow the binding equilibrium to be established, the samples were loaded into MST capillaries and analyzed using a NanoTemper MST instrument. The normalized fluorescence signal (Fnorm) was recorded at each HSA concentration, and the resulting binding curve was fitted using a one-site binding model to determine the apparent equilibrium dissociation constant ($K_{d}$).

### 2.8. Data Processing

Fluorescence images within each comparison group were acquired and processed using identical parameters. Cell viability data were normalized to the untreated control group. All MTT assays were performed in three independent biological experiments, with three technical replicate wells for each concentration in each experiment. Cellular imaging experiments were independently repeated three times. For the quantitative analysis of cellular imaging with the six NI probes, three randomly selected non-overlapping fields were analyzed in each independent experiment, with a total of at least 30 cells analyzed per condition. Data are presented as the mean ± SD of three independent experiments. For HSA-associated cellular imaging, cellular fluorescence was quantified using the probe-channel fluorescence intensity normalized to the corresponding DAPI fluorescence intensity. The resulting probe-to-DAPI fluorescence intensity ratios were expressed as percentages. Data are presented as the mean ± SD (*n* = 5 per condition).

## 3. Results and Discussion

**Scheme 2:**
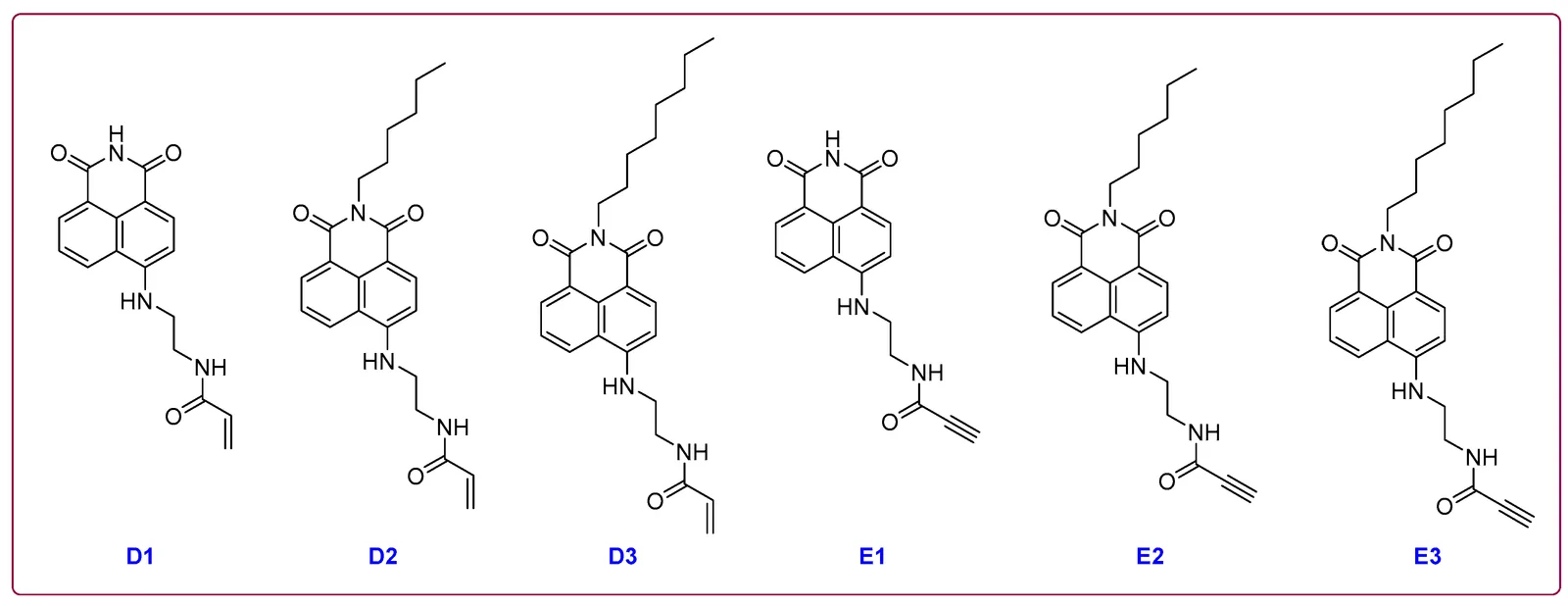
Chemical structure of NI-based fluorescent probes.

Guided by the above design strategy, six NI-based fluorophores (Scheme 2) were synthesized through a three-step procedure described in the Supplementary Materials. First, the imide core was constructed by reacting 4-bromo-1,8-naphthalic anhydride with the corresponding amine, enabling systematic modulation of the N-imide substituent. Second, an ethylenediamine linker was introduced at the brominated aromatic position of the NI backbone, which provided an electron-donating amino substituent and a terminal reactive amine for further derivatization. Finally, the terminal amine was amidated with either acryloyl chloride or propiolic acid to generate alkene-containing acrylamide derivatives (D1–D3) and alkyne-containing propiolamide analogues (E-type probes), respectively. The structures of all intermediates and target compounds were confirmed by NMR spectroscopy (Supplementary Materials). This molecular set was designed to compare two key structure-related parameters: the hydrophobic contribution of the N-imide side chain and the biological effect of alkene versus alkyne terminal unsaturation. Because hydrocarbon substituents may perturb membrane structure and influence cellular physiology [21], subsequent screening focused on both cytocompatibility and cellular fluorescence performance.

**Figure 1:**
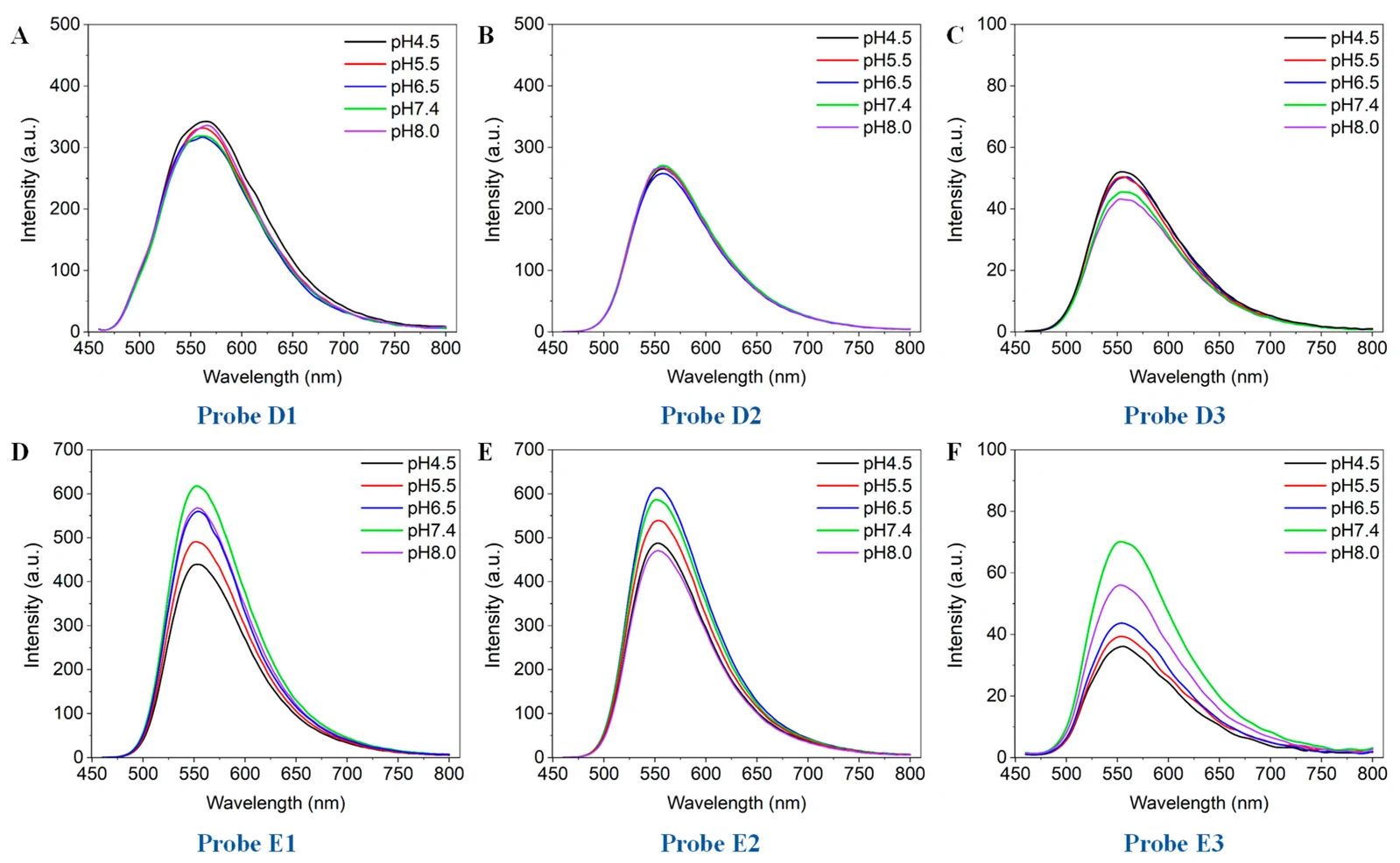
pH-dependent fluorescence emission spectra of (**A**) Probe D1, (**B**) Probe D2, (**C**) Probe D3, (**D**) Probe E1, (**E**) Probe E2, and (**F**) Probe E3 at pH 4.5, 5.5, 6.5, 7.4, and 8.0. Conditions: probe concentration, 5 μM; solvent, PBS containing 1% (*v*/*v*) DMSO and 0.2% (*v*/*v*) Tween 80; λ_ex = 440 nm. For each probe, all pH-dependent spectra were recorded using identical instrumental settings.

The UV–visible absorption spectra of Probe D1–D3 and Probe E1–E3 were recorded under the formulated aqueous conditions described above (Figure S1). All six probes exhibited similar broad absorption bands centered at approximately 435–440 nm. Accordingly, 440 nm was selected as the excitation wavelength for the subsequent fluorescence measurements. The effects of pH on the fluorescence properties of the probes were further evaluated over the pH range of 4.5–8.0 (Figure 1). All six probes retained emission bands centered at approximately 550–560 nm without pronounced shifts in their emission maxima. Probe D1 and Probe D2 showed relatively small intensity variations, whereas Probe D3 exhibited a moderate decrease in fluorescence intensity with increasing pH. In contrast, Probe E1–E3 showed more evident pH-dependent intensity variations, particularly Probe E3. These results indicate that the emission wavelengths were stable across the tested pH range, although the fluorescence intensities, especially those of the E-series probes, were not completely pH-independent. Therefore, pH-dependent intensity changes should be considered when interpreting fluorescence differences in cellular environments.

To quantify the structure-dependent differences in physicochemical properties, ALogP and polar surface area (PSA) values were calculated for the six NI derivatives and Probe D3–ibuprofen (Table 1). Extension of the N-imide alkyl chain progressively increased the calculated lipophilicity within both structural series. The ALogP values increased from 1.458 for D1 to 4.235 for D2 and 5.147 for D3, and from 2.033 for E1 to 4.810 for E2 and 5.722 for E3. In contrast, the calculated PSA values were 90.333 Å^2^ for D1 and E1 and 80.875 Å^2^ for the corresponding N-alkylated derivatives. These results confirm that modification of the N-imide substituent effectively modulated probe lipophilicity.

Calculated lipophilicity did not show a simple monotonic relationship with MTT-based cell viability within this series. Although Probe D3 had a relatively high ALogP value, it retained favorable cell viability, whereas its matched analogue E3 showed lower viability. These comparisons describe the observed physicochemical and cell-viability profiles but do not establish a mechanism for the viability differences.

**Figure 2:**
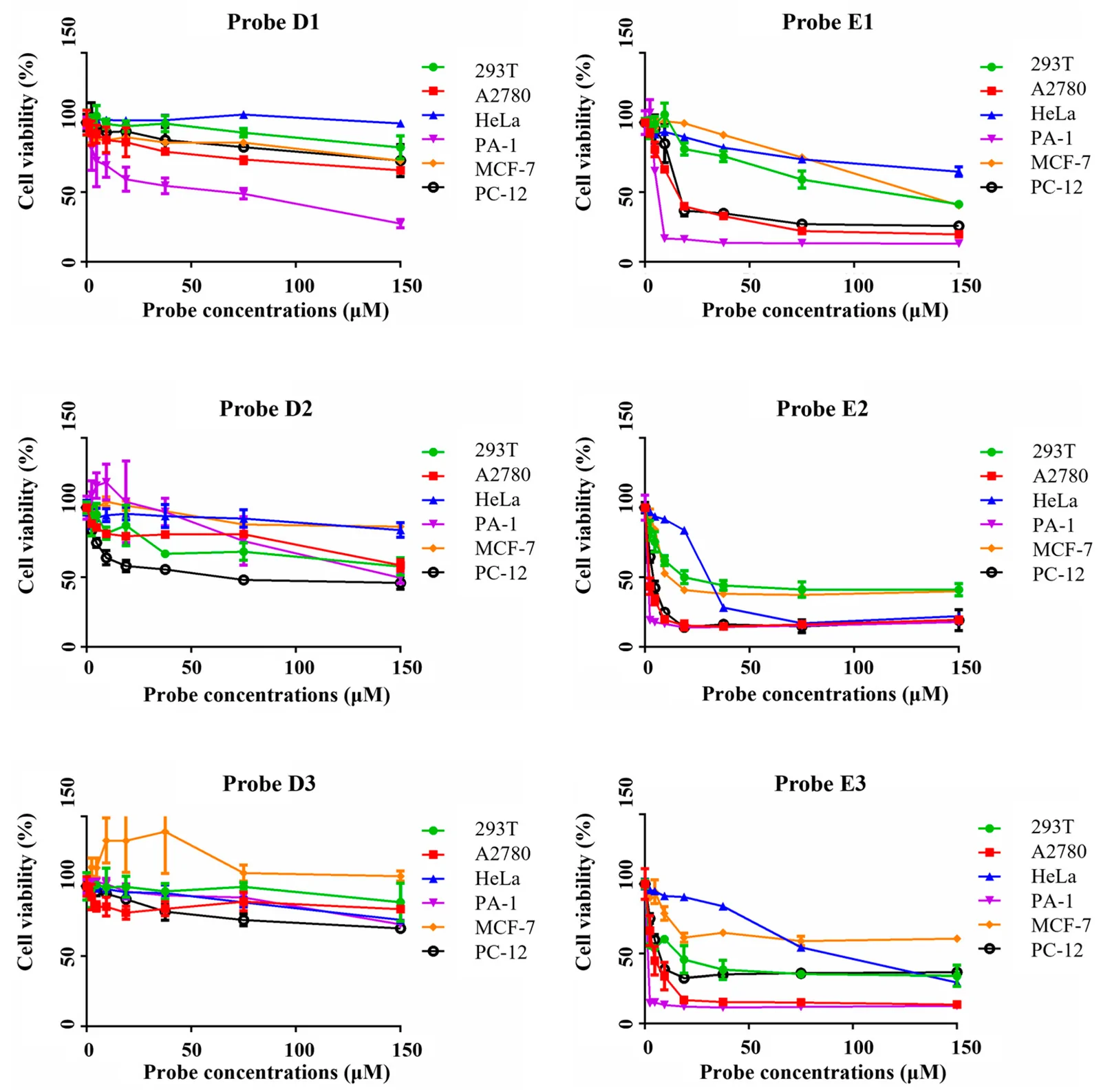
Cytotoxicity evaluation of six NI-based probes in different cell lines using MTT assays. Cells were incubated with the probes at concentrations ranging from 0 to 150 μM for 24 h, followed by MTT treatment for 4 h at 37 °C. Formazan crystals were dissolved in DMSO, and absorbance was measured at 490 nm using a microplate reader. Data are presented as the mean ± SD from three independent biological experiments, each with three technical replicate wells per concentration.

The cell viability profiles of the six candidate probes were evaluated by MTT assays in A2780, HeLa, PA-1, MCF-7, PC-12, and 293T cells. After treatment with probe concentrations of 0–150 μM for 24 h, differences in MTT-based cell viability were observed (Figure 2). Within this six-probe series, the acrylamide-containing probes D1–D3 generally yielded higher cell-viability values than their matched propiolamide analogues E1–E3 under the tested conditions. These results describe an empirical association between terminal unsaturated-amide identity and cell viability; the molecular basis of the differences remains unresolved. Considering both the MTT results and cellular fluorescence performance, Probe D3 was selected as the scaffold for subsequent functionalization.

**Figure 3:**
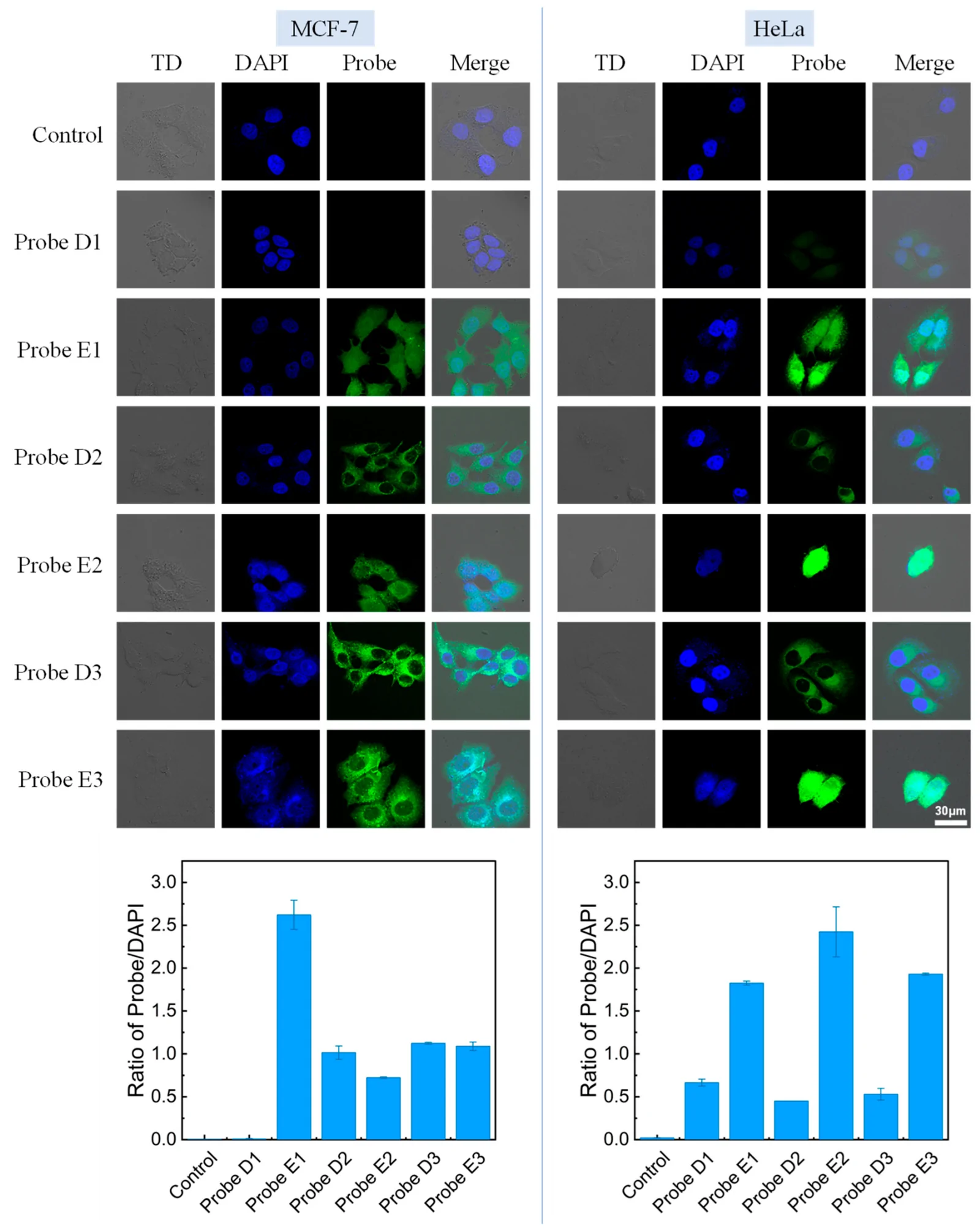
Confocal fluorescence images and quantitative analysis of MCF-7 and HeLa cells incubated with the indicated NI probes (5 μM) for 4 h at 37 °C. From top to bottom, the groups are control, Probe D1, Probe E1, Probe D2, Probe E2, Probe D3, and Probe E3. Transmitted-light (TD), DAPI, probe-fluorescence, and merged images are shown. The DAPI channel was acquired using excitation at 405 nm and emission collection at 420–480 nm. The NI probe channel was acquired using excitation at 445 nm and emission collection at 520–540 nm. Laser power, detector gain, pinhole size, and image-processing parameters were kept identical within each cell-line comparison. The bar graphs show the background-subtracted probe-channel fluorescence intensity normalized to the corresponding DAPI fluorescence intensity. Data are presented as the mean ± SD from three independent biological experiments. Three randomly selected non-overlapping fields were analyzed in each experiment, with a total of at least 30 cells analyzed per condition. Scale bar = 30 μm.

To evaluate the cellular imaging performance of the NI probes, confocal imaging was performed in MCF-7 and HeLa cells, followed by quantitative analysis of the probe-to-DAPI fluorescence intensity ratio (Figure 3). The probes displayed distinct and cell-line-dependent imaging behaviors. Quantitative analysis showed that Probe E1 produced the strongest normalized fluorescence signal in MCF-7 cells, whereas Probe E2 produced the strongest signal in HeLa cells. Probe D3 showed an intermediate but readily detectable signal in both cell lines. The confocal images also suggested differences in the intracellular distribution of the probes. The D-series probes were predominantly localized in the cytoplasmic region and showed limited overlap with the DAPI-stained nuclei. In contrast, some E-series probes, particularly E2 and E3 in HeLa cells, exhibited greater nuclear-associated fluorescence. These images characterize probe fluorescence distribution under the imaging conditions but do not establish the molecular basis of the cell-viability differences. Moreover, fluorescence imaging and MTT assays were performed under different exposure conditions: 5 μM for 4 h and 0–150 μM for 24 h, respectively. Although Probe D3 was not the brightest probe, it retained a readily detectable intracellular signal and exhibited favorable cytocompatibility. Considering both parameters, Probe D3 provided the most favorable overall balance between imaging performance and biocompatibility and was therefore selected as the scaffold for subsequent functionalization.

The green emission of Probe D3 allows spectral separation from the blue DAPI nuclear channel and is compatible with standard confocal microscopy detection settings. However, green-emitting fluorophores do not provide the reduced tissue autofluorescence, lower light scattering, or greater imaging depth generally associated with red- and NIR-emitting probes. Therefore, Probe D3 is suitable for the cultured-cell imaging experiments performed in this study, but its emission wavelength may limit its application in deep-tissue or in vivo imaging. More broadly, recent reviews have highlighted the importance of integrating photophysical tuning, molecular recognition, and biological compatibility in the development of small-molecule probes for disease-oriented bioimaging [22]. In particular, regulation of twisted intramolecular charge transfer (TICT) provides an important framework for designing environment-sensitive fluorescent probes and imaging agents [23]. Together with its favorable cytocompatibility, the optical profile of Probe D3 supports its use as a robust scaffold for developing target-responsive NI probes.

**Figure 4:**
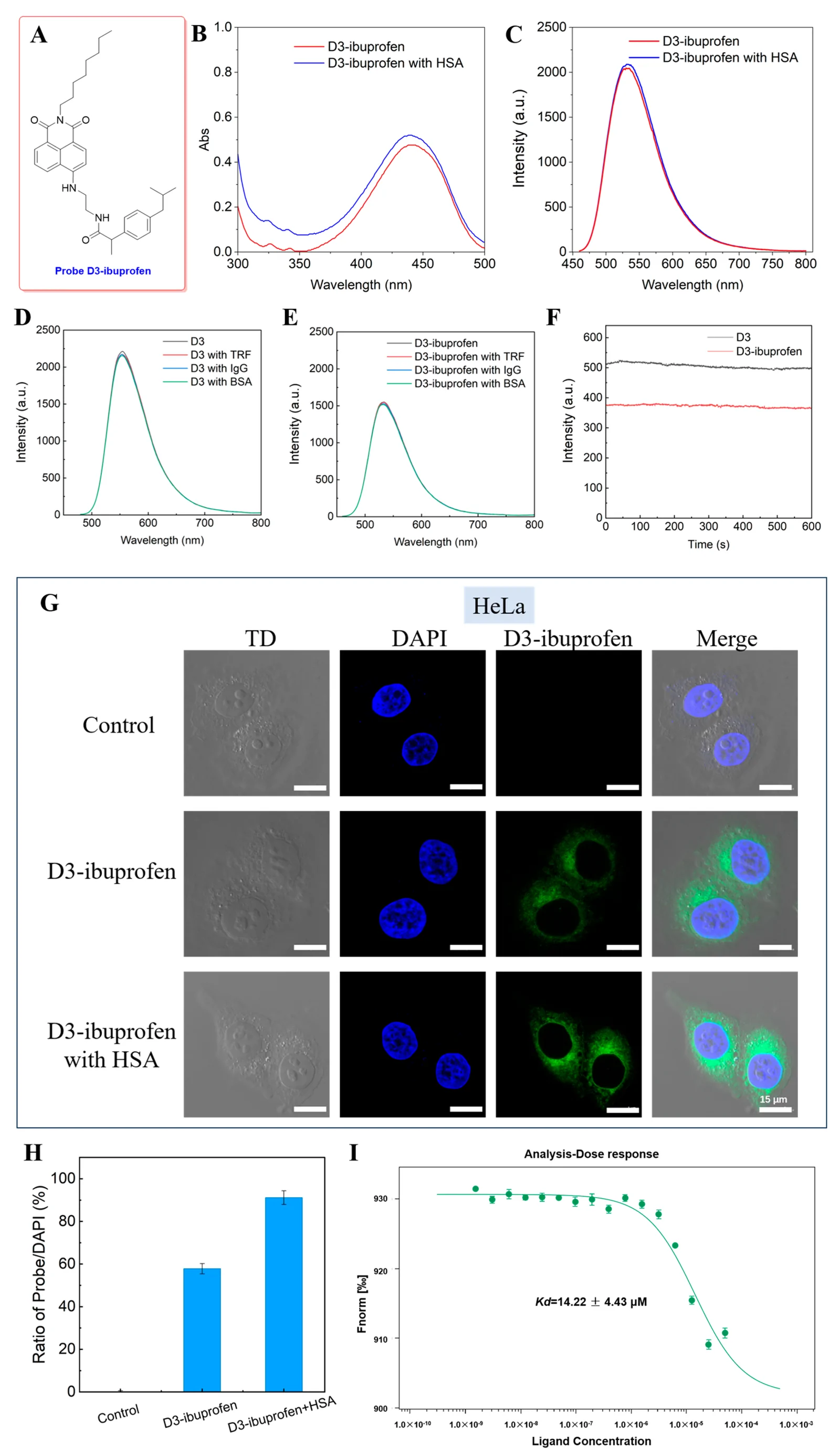
Characterization of HSA binding and cellular imaging by Probe D3–ibuprofen. (**A**) Chemical structure of Probe D3–ibuprofen. (**B**) UV–visible absorption spectra and (**C**) fluorescence emission spectra of Probe D3–ibuprofen in the absence and presence of HSA. The fluorescence spectra were recorded using excitation at 440 nm. (**D**,**E**) Fluorescence emission spectra of (**D**) Probe D3 and (**E**) Probe D3–ibuprofen in the absence and presence of BSA, transferrin (TRF), and IgG. Instrumental settings were identical for all spectra within each comparison. Absolute fluorescence intensities should not be directly compared between panels acquired using different settings. (**F**) Photostability of Probe D3 and Probe D3–ibuprofen measured using a fluorescence spectrometer under continuous excitation at 445 nm for 600 s. Fluorescence emission intensity was continuously monitored at 530 nm. (**G**) Confocal fluorescence images of HeLa cells in the control, Probe D3–ibuprofen, and Probe D3–ibuprofen/HSA groups after incubation for 30 min at 37 °C. The DAPI channel was acquired using excitation at 405 nm and emission collection at 420–480 nm. The probe channel was acquired using excitation at 440 nm and emission collection at 520–540 nm. Identical imaging and processing settings were used for all three groups. The images are representative of three independent biological experiments, with the corresponding fluorescence quantification shown in panel H. Scale bar = 15 μm. [Probe D3–ibuprofen] = 5.0 μM and [HSA] = 10.0 μM. (**H**) Quantitative analysis of the probe-to-DAPI fluorescence intensity ratios for the groups shown in panel G. Ratios are expressed as percentages. Data are presented as the mean ± SD (*n* = 5 per condition). (**I**) MST binding curve obtained by titrating HSA against Probe D3–ibuprofen (200 nM), yielding an apparent Kd value of 14.22 ± 4.43 μM.

To introduce HSA-binding capability into the D3-derived NI scaffold, an ibuprofen-functionalized analogue, Probe D3–ibuprofen, was prepared based on the known binding preference of ibuprofen for Sudlow’s site II of HSA [20] (Figure 4A). HPLC analysis at 440 nm indicated purities of 99.8% and 99.2% for Probe D3 and Probe D3–ibuprofen, respectively (Figures S2 and S3). In the absence of HSA, Probe D3–ibuprofen exhibited a broad absorption band centered at approximately 440 nm and an emission maximum at approximately 535 nm, corresponding to a Stokes shift of approximately 95 nm. Upon addition of HSA, the absorbance around 440 nm increased modestly, accompanied by a slight enhancement in fluorescence intensity around 535 nm (Figure 4B,C). No appreciable shift in either spectral maximum and no new absorption or emission band were observed; therefore, the Stokes shift remained essentially unchanged. These results indicate that HSA association mainly causes a modest intensity modulation, which is consistent with a change in the local environment of the fluorophore rather than a substantial alteration of the electronic structure of the NI chromophore. Because fluorescence quantum yield and lifetime were not determined, a more detailed excited-state mechanism cannot be assigned from the present data.

Ibuprofen derivatization markedly altered the calculated physicochemical profile of the probe. Compared with Probe D3, Probe D3–ibuprofen showed an increase in ALogP from 5.147 to 8.261, whereas its calculated PSA remained unchanged at 80.875 Å^2^ (Table 1). These results indicate that introduction of the ibuprofen moiety substantially increased the predicted lipophilicity of the probe without altering its calculated polar surface area. This increased lipophilicity may affect its association with hydrophobic protein-binding pockets and its cellular distribution. However, ALogP and PSA are computational descriptors and do not directly demonstrate aqueous solubility, aggregation behavior, or chemical stability. Therefore, no conclusion regarding improved solubility or stability was drawn solely from these calculations. MST analysis confirmed an interaction between Probe D3–ibuprofen and HSA, with an apparent Kd of 14.22 ± 4.43 μM (Figure 4I). In the cellular imaging experiment, control HeLa cells showed negligible green fluorescence, whereas cells treated with Probe D3–ibuprofen displayed a clear cellular fluorescence signal (Figure 4G). Quantitative image analysis showed a higher probe-to-DAPI fluorescence intensity ratio in the Probe D3–ibuprofen/HSA group than in the probe-only group (Figure 4H). This analysis supports the observed cellular fluorescence difference but does not directly quantify HSA concentration or the amount of internalized probe.

In addition, because HSA induced only modest changes in the solution fluorescence and no concentration-dependent analytical calibration curve was established, a reliable limit of detection could not be determined. The present results therefore demonstrate the binding of Probe D3–ibuprofen to HSA and its potential for HSA-associated cellular imaging, rather than quantitative determination of HSA.

The present study was designed as a proof-of-concept investigation of HSA-associated sensing in human-derived cells rather than as a cross-species albumin-selectivity study. To assess the effects of other proteins on probe fluorescence, the emission spectra of Probe D3 and Probe D3–ibuprofen were examined in the presence of BSA, transferrin (TRF), and IgG (Figure 4D,E). These proteins caused only small changes in the fluorescence spectra under the tested conditions. However, limited fluorescence modulation does not exclude protein binding, and these measurements do not establish preferential binding to HSA. Together with the MST results, the present findings support the interaction of Probe D3–ibuprofen with HSA and its potential for HSA-associated cellular imaging, while its binding selectivity over other proteins remains to be established.

The photostability of Probe D3 and Probe D3–ibuprofen was further evaluated under continuous excitation at 440 nm, with fluorescence emission monitored at 530 nm for 600 s (Figure 4F). Both probes showed only small decreases in fluorescence intensity over the monitoring period, indicating limited fluorescence decay under the tested excitation conditions. The higher cellular fluorescence signal may reflect HSA-associated cellular uptake, a protein-induced change in probe emission, or both. Related albumin-assisted probe systems provide relevant context for this interpretation [24]. Prebinding a naphthalimide-containing photochromic probe to HSA was reported to enhance its aqueous fluorescence, solubility, and cellular permeability [25], while BSA encapsulation and albumin-based supramolecular assembly have enabled long-wavelength lipid-droplet imaging in living cells and mouse models of fatty liver [26,27]. Unlike solution measurements at a fixed probe concentration, cellular fluorescence reflects both local probe abundance and probe brightness. HSA association may affect probe delivery, accumulation, or retention, while the cellular microenvironment may also modulate probe emission. The present data do not distinguish the relative contributions of these processes. Therefore, the stronger cellular fluorescence cannot be attributed solely to the modest fluorescence enhancement observed in solution, and HSA-facilitated probe internalization remains a possible explanation rather than an experimentally established mechanism.

## 4. Conclusions

In summary, a chain-length-optimized NI fluorescent probe platform was developed by systematically tuning the N-imide alkyl substituent and the terminal unsaturated amide group. Within this six-probe series, the acrylamide-containing D probes generally exhibited higher MTT-based cell viability than their matched propiolamide-containing E analogues. Probe D3, bearing an octyl imide chain, displayed the most favorable combination of low cytotoxicity and readily detectable intracellular fluorescence. Probe D3 emitted in the green region, with an emission maximum at approximately 550–560 nm, supporting its use in confocal cellular imaging. Following conjugation with ibuprofen, Probe D3–ibuprofen retained a similar absorption and emission profile, showed modest HSA-induced spectral changes, and interacted with HSA with an apparent K_d_ value of 14.22 ± 4.43 μM, as determined by MST. Quantitative image analysis showed higher normalized cellular fluorescence in the Probe D3–ibuprofen/HSA group than in the probe-only group. These findings support the integration of chain-length optimization and ligand-directed functionalization for protein-associated cellular imaging. The HSA-associated imaging results remain proof-of-concept evidence and do not establish quantitative HSA detection or HSA-facilitated probe internalization. Both Probe D3 and Probe D3–ibuprofen showed limited fluorescence decay during 600 s of continuous excitation under the tested conditions. Further comparative protein-binding studies and evaluation in more physiologically relevant biological models will be required to establish the selectivity and broader applicability of Probe D3–ibuprofen.

## Figures and Tables

**Table 1:** Calculated physicochemical descriptors of the NI-based probes and Probe D3-ibuprofen.

Probe	AlogP ^[a]^	PSA (Å^2^) ^[a]^
D1	1.458	90.333
D2	4.235	80.875
D3	5.147	80.875
E1	2.033	90.333
E2	4.810	80.875
E3	5.722	80.875
D3–ibuprofen	8.261	80.875

[a] Calculated using BIOVIA Discovery Studio 2024. ALogP is dimensionless, and PSA is expressed in Å^2^.

## Data Availability

The data supporting the findings of this study are available from the corresponding authors upon reasonable request.

## Supplementary Materials

The following supporting information can be downloaded at: https://www.mdpi.com/article/10.3390/s26216702/s1, synthetic procedures and characterization data; ^1^H NMR spectra of the synthesized compounds; HPLC analysis conditions; Figure S1: UV–visible absorption spectra of Probe D1–D3 and Probe E1–E3; Figure S2: HPLC chromatogram of Probe D3 recorded at a detection wavelength of 440 nm; Figure S3: HPLC chromatogram of Probe D3–ibuprofen recorded at a detection wavelength of 440 nm.
