# Supplementary material for: Chain-Length-Optimized 1,8-Naphthalimide–Ibuprofen Fluorescent Probe for HSA Binding and Cellular Imaging
Source: Sensors (Basel). 2026 Oct 23;26(21):6702. doi: 10.3390/s26216702 (PMC13766202; doi:10.3390/s26216702)
Supplement: Supplementary file 1 [file sensors-26-06702-s001.zip › sensors-4451199-supplementary.pdf]

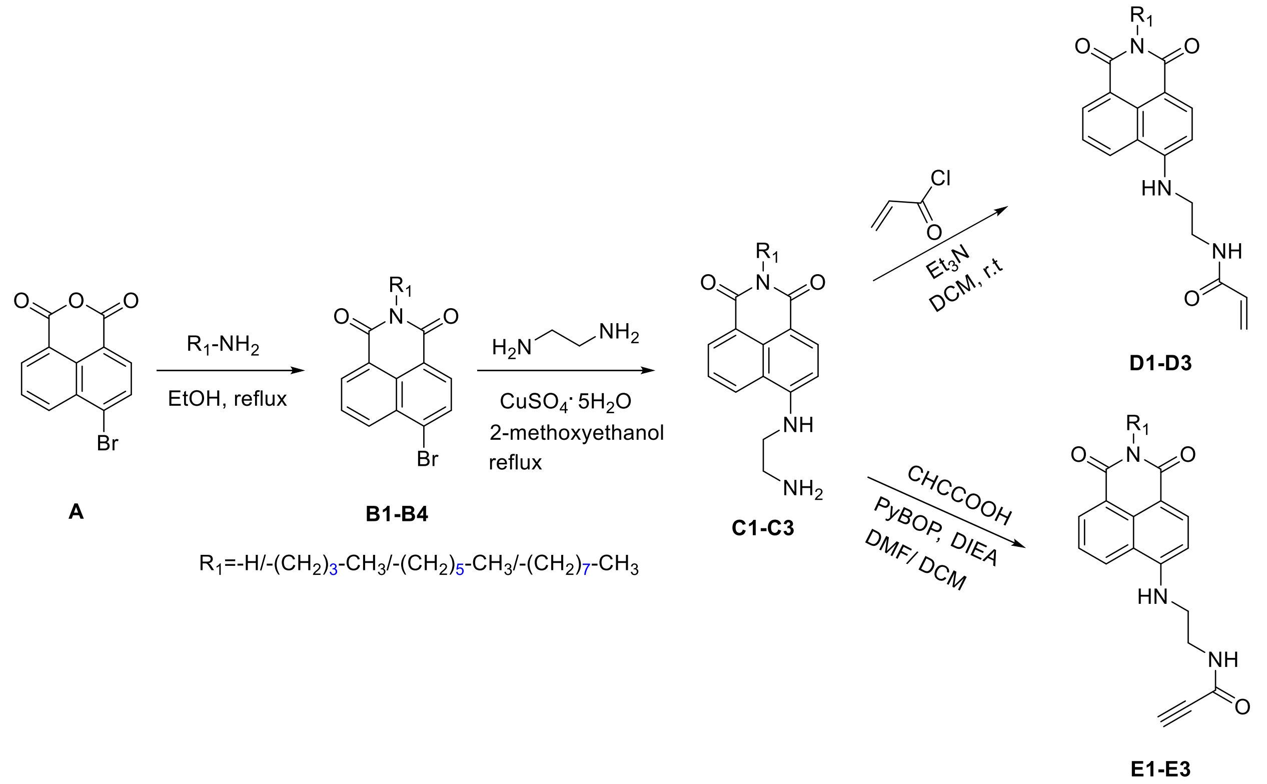

1. General procedure for the synthesis of compound **B**

4-Bromo-1,8-naphthalic anhydride (2.7 g, 10 mmol) and the appropriately substituted amine (12 mmol) were added to ethanol (300 mL). The mixture was heated to reflux and stirred overnight. After completion, the reaction was cooled to room temperature, and the resulting precipitate was collected by filtration and washed with ethanol to yield a brown solid. The crude product was recrystallized from an appropriate volume of acetic acid to afford the pure target compound **B**.

6-bromo-1*H*-benzo[*de*]isoquinoline-1,3(2*H*)-dione (**B1**): Brown solid, yield 87%. ^{1}H NMR (400 MHz, DMSO-d_6) δ 11.79 (s, 1H), 8.49 (t, *J* = 7.4 Hz, 2H), 8.24 (d, *J* = 7.8 Hz, 1H), 8.16 (d, *J* = 7.8 Hz, 1H), 7.95 (t, *J* = 7.9 Hz, 1H).

6-bromo-2-hexyl-1H-benzo[de]isoquinoline-1,3(2H)-dione (**B2**): Brown solid, yield 79%. ^{1}H NMR (400 MHz, DMSO-d_6) δ 8.53 (dd, *J* = 12.0, 7.9 Hz, 2H), 8.31 (dd, *J* = 7.9, 1.2 Hz, 1H), 8.20 (dd, *J* = 7.9, 1.2 Hz, 1H), 8.03 – 7.94 (m, 1H), 4.01 (t, *J* = 7.5 Hz, 2H), 1.62 (p, *J* = 7.4 Hz, 2H), 1.40 – 1.23 (m, 6H), 0.92 – 0.81 (m, 3H).

6-bromo-2-octyl-1H-benzo[de]isoquinoline-1,3(2H)-dione (**B3**): Brown solid, yield 81%. ^{1}H NMR (400 MHz, DMSO-d_6) δ 8.56 (dd, *J* = 10.2, 7.9 Hz, 2H), 8.33 (d, *J* = 7.8 Hz, 1H), 8.22 (d, *J* = 7.9 Hz, 1H), 8.00 (t, *J* = 7.9 Hz, 1H), 4.02 (t, *J* = 7.5 Hz, 2H), 1.63 (t, *J* = 7.3 Hz, 2H), 1.39 – 1.16 (m, 10H), 0.84 (d, *J* = 7.1 Hz, 3H).

2. General Procedure for the Synthesis of Compounds **C**

To a stirred solution of Compound **B** (10.0 mmol, 1.0 equiv.) in 2-methoxyethanol (50 mL) were added ethylenediamine (0.1 mol, 10.0 equiv.) and $CuSO_4.5H_2O$ (1.0 mmol, 0.1 equiv.). The reaction mixture was heated to reflux and maintained for 12 h under a nitrogen atmosphere. The progress of the reaction was monitored by TLC. Upon completion, the mixture was cooled to room temperature and poured into 200 mL of ice-water with vigorous stirring. The resulting orange-red precipitate was collected by filtration and washed several times with distilled water to remove excess amine and copper residues. The crude product was dried under vacuum and further purified by silica gel column chromatography to afford the target compound C.

6-((2-aminoethyl)amino)-1*H*-benzo[*de*]isoquinoline-1,3(2*H*)-dione (**C1**): Yellow solid, yield 83%. ^{1}H NMR (400 MHz, DMSO-d_6) δ 8.68 (d, *J* = 8.4 Hz, 1H), 8.36 (d, *J* = 7.2 Hz, 1H), 8.18 (d, *J* = 8.5 Hz, 1H), 7.64 (dd, *J* = 8.4, 7.3 Hz, 1H), 6.77 (d, *J* = 8.6 Hz, 1H), 3.35 (d, *J* = 6.7 Hz, 4H), 2.85 (s, 2H).

6-((2-aminoethyl)amino)-2-hexyl-1*H*-benzo[*de*]isoquinoline-1,3(2*H*)-dione (**C2**): Yellow solid, yield 80%. ^{1}H NMR (400 MHz, DMSO-d_6) δ 8.72 (d, *J* = 8.4 Hz, 1H), 8.42 (d, *J* = 7.3 Hz, 1H), 8.25 (d, *J* = 8.4 Hz, 1H), 7.68 (t, *J* = 7.8 Hz, 1H), 6.81 (d, *J* = 8.6 Hz, 1H), 4.00 (t, *J* = 7.5 Hz, 2H), 3.39 (t, *J* = 6.5 Hz, 3H), 2.89 (s, 2H), 1.59 (q, *J* = 7.3 Hz, 2H), 1.30 (s, 7H), 0.86 (d, *J* = 6.5 Hz, 3H).

6-((2-aminoethyl)amino)-2-octyl-1*H*-benzo[*de*]isoquinoline-1,3(2*H*)-dione (**C3**): Yellow solid, yield 78%. ^{1}H NMR (400 MHz, DMSO-d_6) δ 8.71 (d, *J* = 8.4 Hz, 1H), 8.42 (d, *J* = 7.3 Hz, 1H), 8.25 (d, *J* = 8.4 Hz, 1H), 7.67 (t, *J* = 7.8 Hz, 1H), 6.80 (d, *J* = 8.6 Hz, 1H), 3.99 (t, *J* = 7.5 Hz, 2H), 3.56 – 3.37 (m, 2H), 1.58 (q, *J* = 7.3 Hz, 2H), 1.38 – 1.15 (m, 10H), 0.91 – 0.78 (m, 3H).

3. General Procedure for the Synthesis of Acrylamide Derivatives **D**

To a stirred solution of Compound **C** (5.0 mmol, 1.0 equiv.) and triethylamine (1.4 mL, 10.0 mmol, 2.0 equiv.) in anhydrous dichloromethane (30 mL) at 0 °C (ice-water bath) under a nitrogen atmosphere, acryloyl chloride (0.49 mL, 6.0 mmol, 1.2 equiv.) was added dropwise over 10 minutes. Following the addition, the ice bath was removed, and the reaction mixture was allowed to warm to room temperature and stirred for an additional 4–6 h. The progress of the reaction was monitored by TLC. Upon completion, the reaction mixture was quenched with the addition of saturated aqueous $NaHCO_3$ (30 mL). The organic layer was separated, and the aqueous phase was extracted with dichloromethane. The combined organic layers were washed sequentially with

distilled water (30 mL) and brine (30 mL), dried over anhydrous Na_2SO_4, and filtered. The solvent was removed under reduced pressure, and the resulting crude residue was purified by silica gel column chromatography to afford the pure polymerizable target compound **D**.

N-(2-((1,3-dioxo-2,3-dihydro-1*H*-benzo[*de*]isoquinolin-6-yl)amino)ethyl)acrylamide (**D1**): Yellow solid, yield 88%. ^{1}H NMR (400 MHz, DMSO-d$_6$) δ 10.34 (s, 1H), 8.69 (d, *J* = 8.5 Hz, 1H), 8.56 (d, *J* = 5.7 Hz, 1H), 8.36 (d, *J* = 7.2 Hz, 1H), 8.18 (d, *J* = 8.5 Hz, 1H), 7.97 (d, *J* = 4.9 Hz, 1H), 7.66 (t, *J* = 7.9 Hz, 1H), 6.83 (d, *J* = 8.6 Hz, 1H), 6.23 (dd, *J* = 17.1, 10.0 Hz, 1H), 6.11 (dd, *J* = 17.1, 2.4 Hz, 1H), 5.59 (dd, *J* = 10.0, 2.4 Hz, 1H), 3.46 (dt, *J* = 10.3, 5.6 Hz, 4H), 3.02 (q, *J* = 7.3 Hz, 4H).

N-(2-((2-hexyl-1,3-dioxo-2,3-dihydro-1*H*-benzo[*de*]isoquinolin-6-yl)amino)ethyl)acrylamide (**D2**): Yellow solid, yield 71%. ^{1}H NMR (400 MHz, DMSO-d$_6$) δ 8.62 (dd, *J* = 8.5, 1.1 Hz, 1H), 8.47 – 8.43 (m, 1H), 8.41 (d, *J* = 12.4 Hz, 1H), 8.26 (d, *J* = 8.5 Hz, 1H), 7.88 (s, 1H), 7.70 (dd, *J* = 8.4, 7.3 Hz, 1H), 6.87 (d, *J* = 8.6 Hz, 1H), 6.23 (dd, *J* = 17.1, 9.9 Hz, 1H), 6.13 (dd, *J* = 17.1, 2.4 Hz, 1H), 5.63 (dd, *J* = 9.9, 2.4 Hz, 1H), 4.05 – 3.96 (m, 2H), 3.48 (d, *J* = 8.7 Hz, 4H), 1.59 (d, *J* = 8.0 Hz, 2H), 1.41 – 1.22 (m, 6H), 0.95 – 0.77 (m, 3H).

N-(2-((2-octyl-1,3-dioxo-2,3-dihydro-1*H*-benzo[*de*]isoquinolin-6-yl)amino)ethyl)acrylamide (**D3**): Yellow solid, yield 76%. ^{1}H NMR (400 MHz, DMSO-d$_6$) δ 8.62 (d, *J* = 8.4 Hz, 1H), 8.46 – 8.42 (m, 1H), 8.40 (d, *J* = 5.8 Hz, 1H), 8.26 (d, *J* = 8.5 Hz, 1H), 7.88 (d, *J* = 5.8 Hz, 1H), 7.70 (dd, *J* = 8.4, 7.3 Hz, 1H), 6.87 (d, *J* = 8.6 Hz, 1H), 6.23 (dd, *J* = 17.1, 9.9 Hz, 1H), 6.13 (dd, *J* = 17.1, 2.4 Hz, 1H), 5.63 (dd, *J* = 9.9, 2.5 Hz, 1H), 4.00 (t, *J* = 7.4 Hz, 2H), 3.48 (dd, *J* = 7.0, 4.0 Hz, 4H), 1.59 (s, 2H), 1.36 – 1.17 (m, 10H), 0.89 – 0.80 (m, 3H).

4. General Procedure for the Synthesis of Propiolamide Derivatives **E**

To a solution of compound C (0.001 mol), propiolic acid (75 µl, 0.0012 mol) and PyBOP (631mg, 0.0012 mol) in anhydrous DMF (2 ml) and dichloromethane (5 ml) was added DIEA (890 µl, 0.005 mol) at room temperature. After being stirred overnight, the reaction mixture was extracted with ethyl acetate (100 ml), washed with saturated NH_4Cl aq and brine, dried over Na_2SO_4, evaporated under reduced pressure. Purification by silica-gel chromatography yield the product.

N-(2-((1,3-dioxo-2,3-dihydro-1H-benzo[de]isoquinolin-6-yl)amino)ethyl)propiolamide (**E1**): Yellow solid, yield 78%. ^{1}H NMR (400 MHz, DMSO-d$_6$) δ 10.34 (s, 1H), 8.93 (t, *J* = 5.7 Hz, 1H), 8.69 (d, *J* = 8.5 Hz, 1H), 8.36 (d, *J* = 7.3 Hz, 1H), 8.18 (d, *J* = 8.5 Hz, 1H), 7.95 (t, *J* = 5.6 Hz, 1H), 7.66 (t, *J* = 7.9 Hz, 1H), 6.83 (d, *J* = 8.6 Hz, 1H), 4.18 (s, 1H), 3.46 (m, 4H).

N-(2-((2-hexyl-1,3-dioxo-2,3-dihydro-1H-benzo[de]isoquinolin-6-yl)amino)ethyl)propiolamide (**E2**): Yellow solid, yield 69%. ^{1}H NMR (400 MHz, DMSO-d_6) δ 8.93 (t, *J* = 5.7 Hz, 1H), 8.62 (dd, *J* = 8.6, 1.2 Hz, 1H), 8.44 (dd, *J* = 7.3, 1.0 Hz, 1H), 8.26 (d, *J* = 8.5 Hz, 1H), 7.80 (t, *J* = 5.6 Hz, 1H), 7.70 (dd, *J* = 8.4, 7.3 Hz, 1H), 6.84 (d, *J* = 8.6 Hz, 1H), 4.18 (s, 1H), 4.00 (dd, *J* = 8.5, 6.4 Hz, 2H), 3.48 (t, *J* = 5.8 Hz, 2H), 3.42 (q, *J* = 6.3 Hz, 2H), 1.59 (q, *J* = 7.2 Hz, 2H), 1.38 – 1.22 (m, 7H), 0.91 – 0.81 (m, 3H).

N-(2-((2-octyl-1,3-dioxo-2,3-dihydro-1H-benzo[de]isoquinolin-6-yl)amino)ethyl)propiolamide (**E3**): Yellow solid, yield 64%. ^{1}H NMR (400 MHz, DMSO-d_6) δ 8.93 (t, *J* = 5.7 Hz, 1H), 8.62 (dd, *J* = 8.7, 1.1 Hz, 1H), 8.44 (dd, *J* = 7.3, 1.0 Hz, 1H), 8.26 (d, *J* = 8.5 Hz, 1H), 7.81 (t, *J* = 5.6 Hz, 1H), 7.70 (dd, *J* = 8.4, 7.3 Hz, 1H), 6.84 (d, *J* = 8.7 Hz, 1H), 4.18 (s, 1H), 4.04 – 3.96 (m, 2H), 3.53 – 3.46 (m, 2H), 3.46 – 3.38 (m, 2H), 1.64 – 1.55 (m, 2H), 1.36 – 1.18 (m, 11H), 0.89 – 0.81 (m, 3H).

5. Procedure for the Synthesis of Probe **D3–ibuprofen**

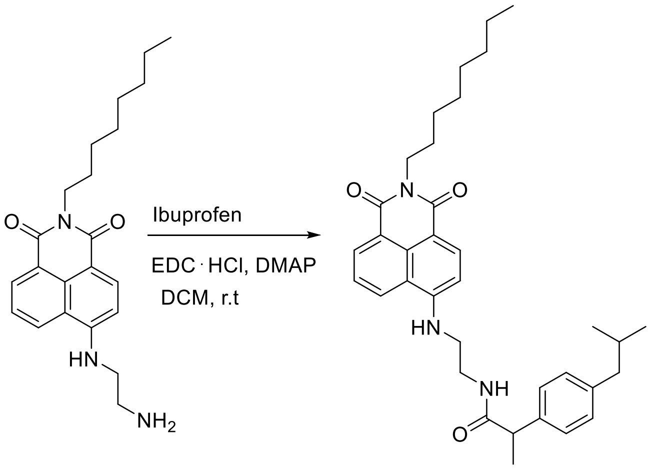

To a stirred solution of compound C3 (0.001 mol, 1.0 equiv.) and ibuprofen (247 mg, 1.2 mmol, 1.2 equiv.) in anhydrous dichloromethane (15 mL) at room temperature, 1-ethyl-3-(3-dimethylaminopropyl)carbodiimide hydrochloride (EDC·HCl, 288 mg, 1.5 mmol, 1.5 equiv.) and 4-dimethylaminopyridine (DMAP, 146 mg, 1.2 mmol, 1.2 equiv.) were added sequentially. The reaction mixture was stirred at room temperature for 12 h under a nitrogen atmosphere. The progress of the reaction was monitored by TLC. Upon completion, the reaction mixture was diluted with additional dichloromethane (20 mL) and washed sequentially with saturated aqueous NH_4Cl (20 mL), saturated aqueous $NaHCO_3$ (20 mL), and brine (20 mL). The organic layer was dried over anhydrous Na_2SO_4, filtered, and evaporated under reduced pressure. The resulting crude

residue was purified by silica gel column chromatography to afford the pure ibuprofen-conjugated target compound.

2-(4-isobutylphenyl)-*N*-(2-((2-octyl-1,3-dioxo-2,3-dihydro-1*H*-benzo[*de*]isoquinolin-6-yl)amino) ethyl)propanamide (**D3–ibuprofen**): Yellow solid, yield 58%. ^{1}H NMR (400 MHz, DMSO-d_6) δ 8.54 (dd, *J* = 8.6, 1.2 Hz, 1H), 8.44 (dd, *J* = 7.4, 1.0 Hz, 1H), 8.26 – 8.16 (m, 2H), 7.74 – 7.64 (m, 2H), 7.19 – 7.12 (m, 2H), 6.97 – 6.91 (m, 2H), 6.80 (d, *J* = 8.6 Hz, 1H), 4.00 (dd, *J* = 8.3, 6.6 Hz, 2H), 3.54 (q, *J* = 7.0 Hz, 1H), 3.47 – 3.35 (m, 4H), 2.31 (d, *J* = 7.1 Hz, 2H), 1.68 (dt, *J* = 13.5, 6.8 Hz, 1H), 1.64 – 1.55 (m, 2H), 1.36 – 1.20 (m, 13H), 0.89 – 0.81 (m, 3H), 0.77 (dd, *J* = 6.6, 0.9 Hz, 6H).

6. HPLC Analysis of Probe D3 and Probe D3–ibuprofen

HPLC analysis was performed using an Agilent 1260 HPLC system equipped with a diode array detector (DAD). Chromatographic separation was achieved on an Agilent ZORBAX Eclipse Plus C18 column (150 × 4.6 mm, 5 μm) maintained at 30 °C. The mobile phase consisted of water containing 0.1% (v/v) formic acid (A) and methanol (B). Isocratic elution was performed at an A/B ratio of 60:40 (v/v), with a flow rate of 1.0 mL min^{-1}. The injection volume was 10.0 μL, and the detection wavelength was set at 440 nm. The total run time was 40 min.

^{1}H NMR spectrum of compound B1

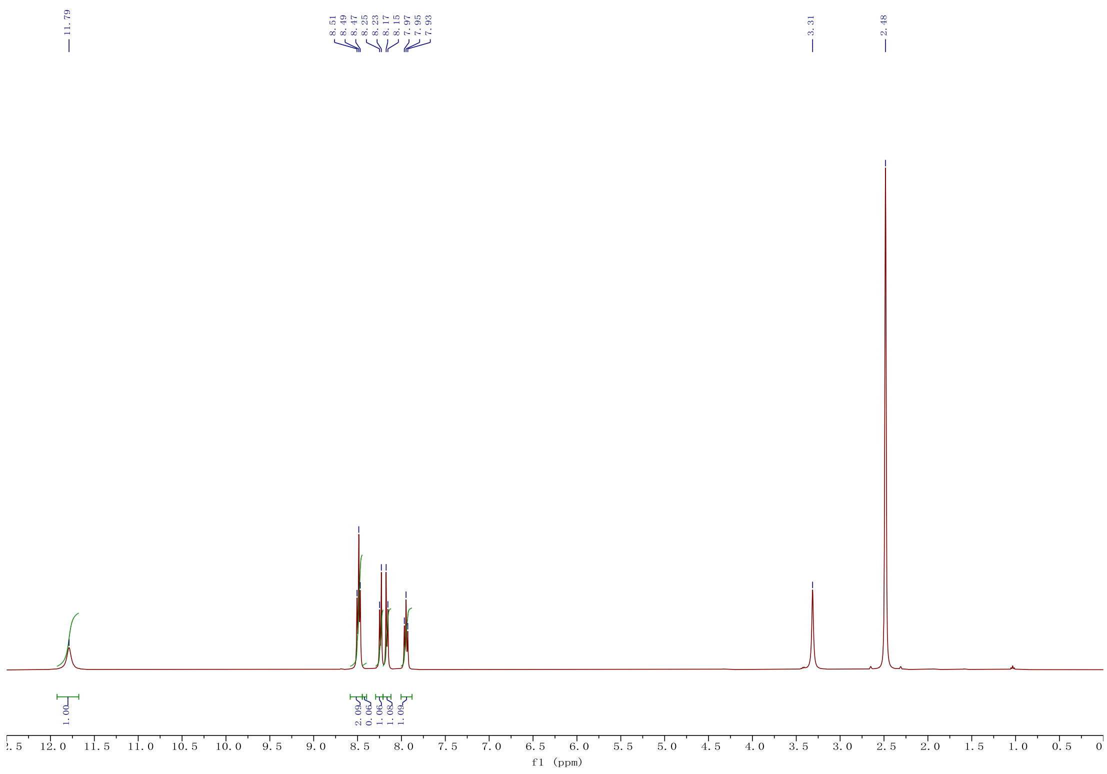

¹H NMR spectrum of compound B2

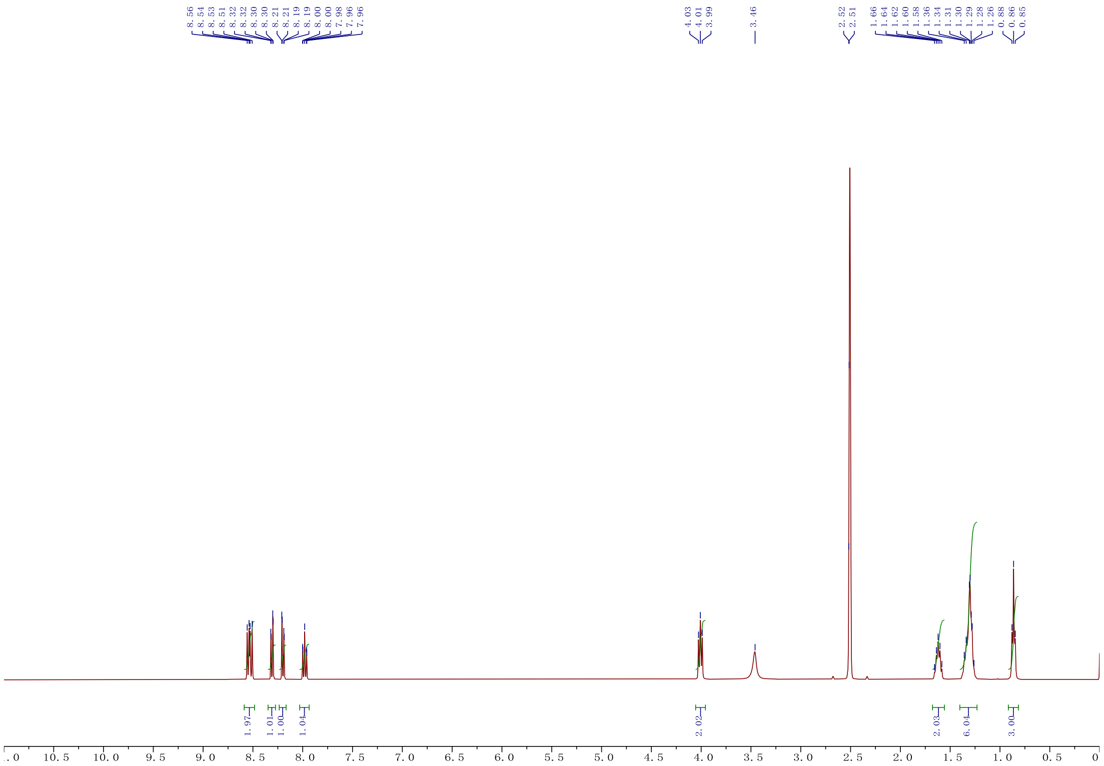

¹H NMR spectrum of compound B3

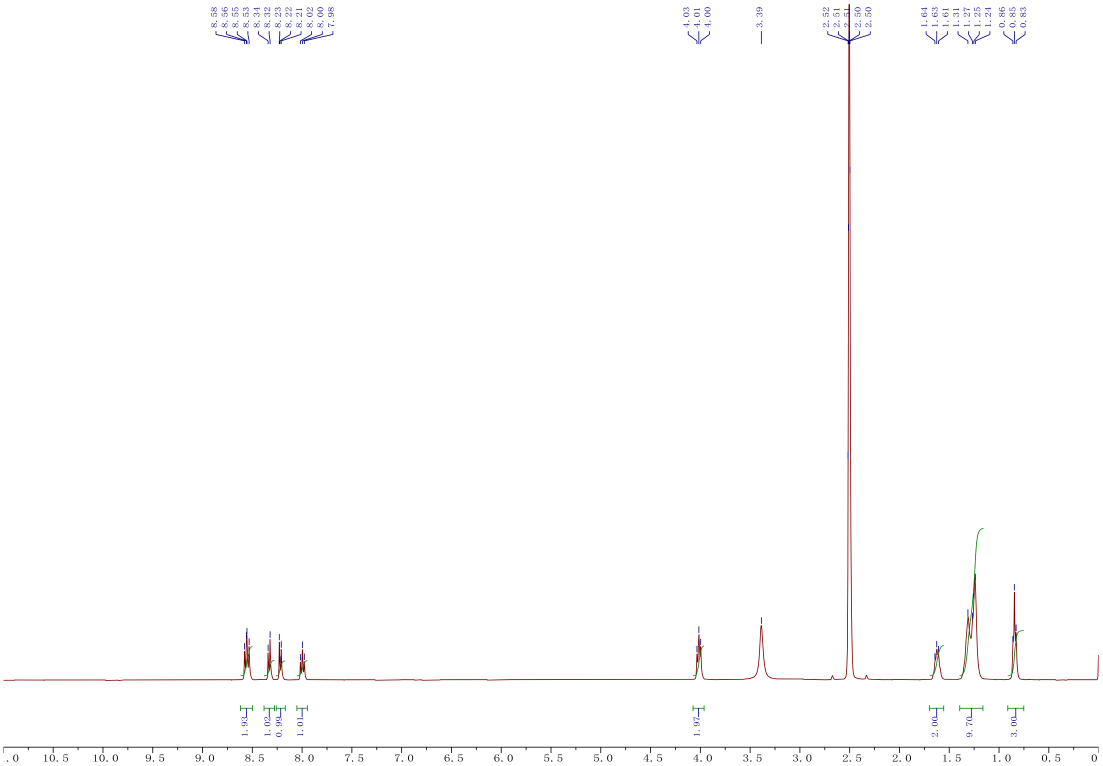

^{1}H NMR spectrum of compound C1

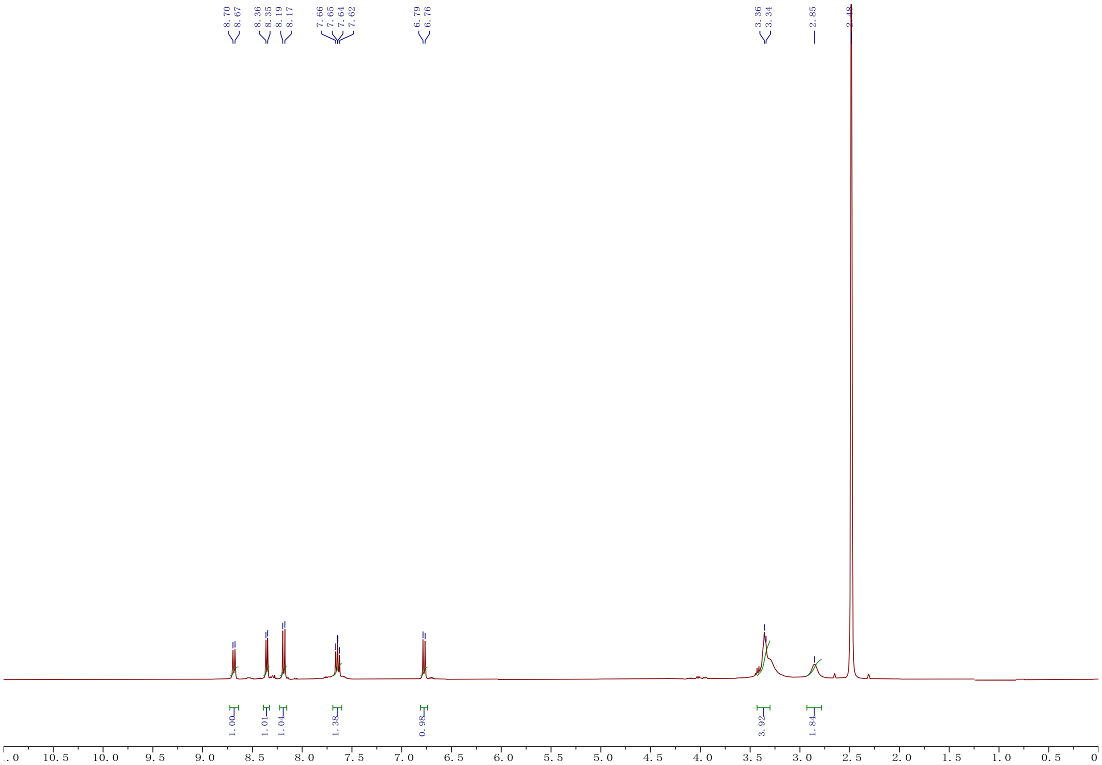

^{1}H NMR spectrum of compound C2

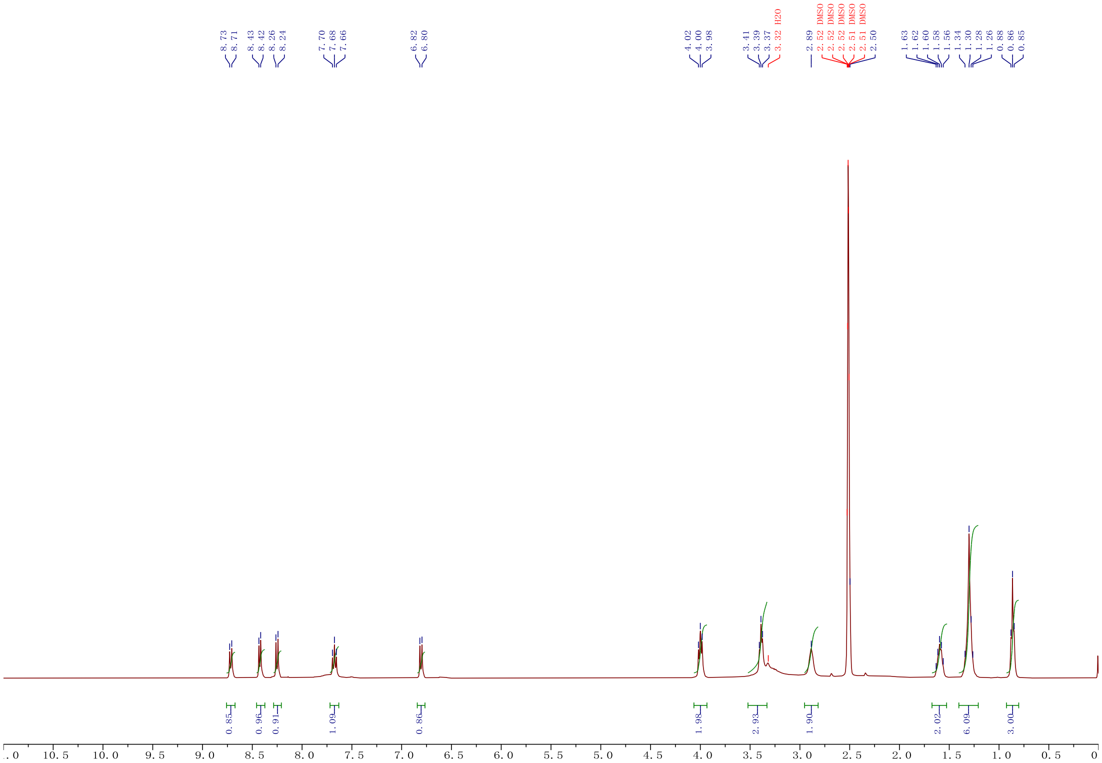

¹H NMR spectrum of compound C3

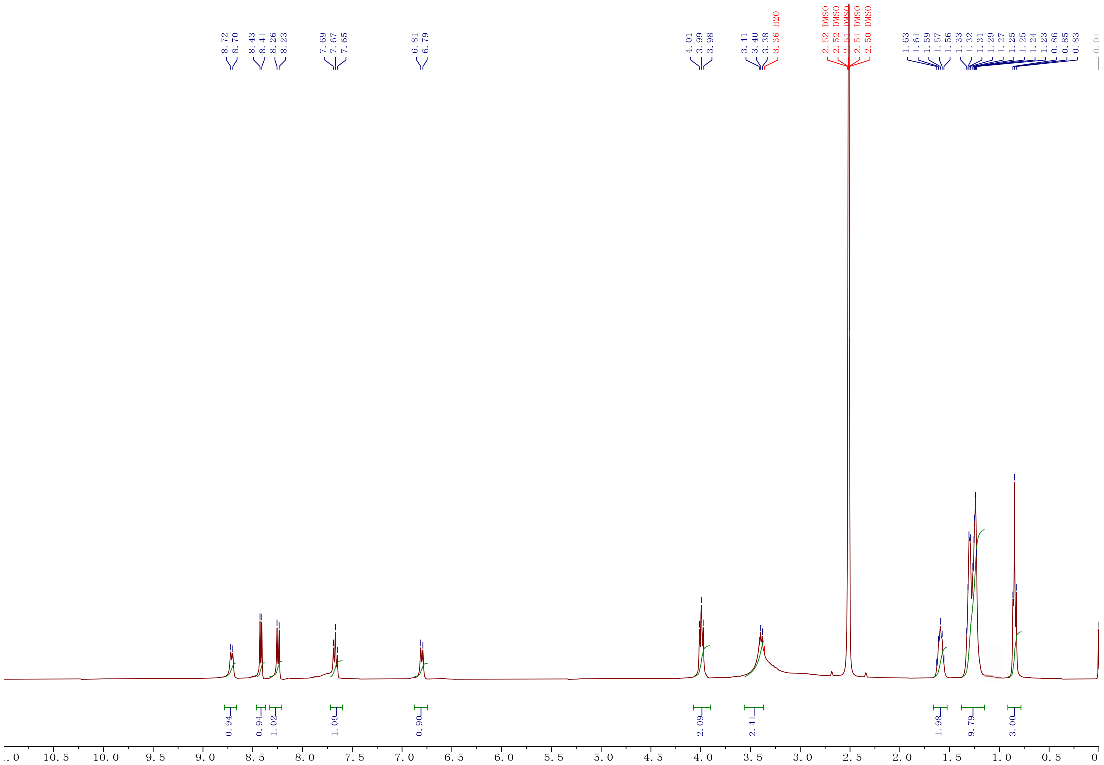

¹H NMR spectrum of compound D1

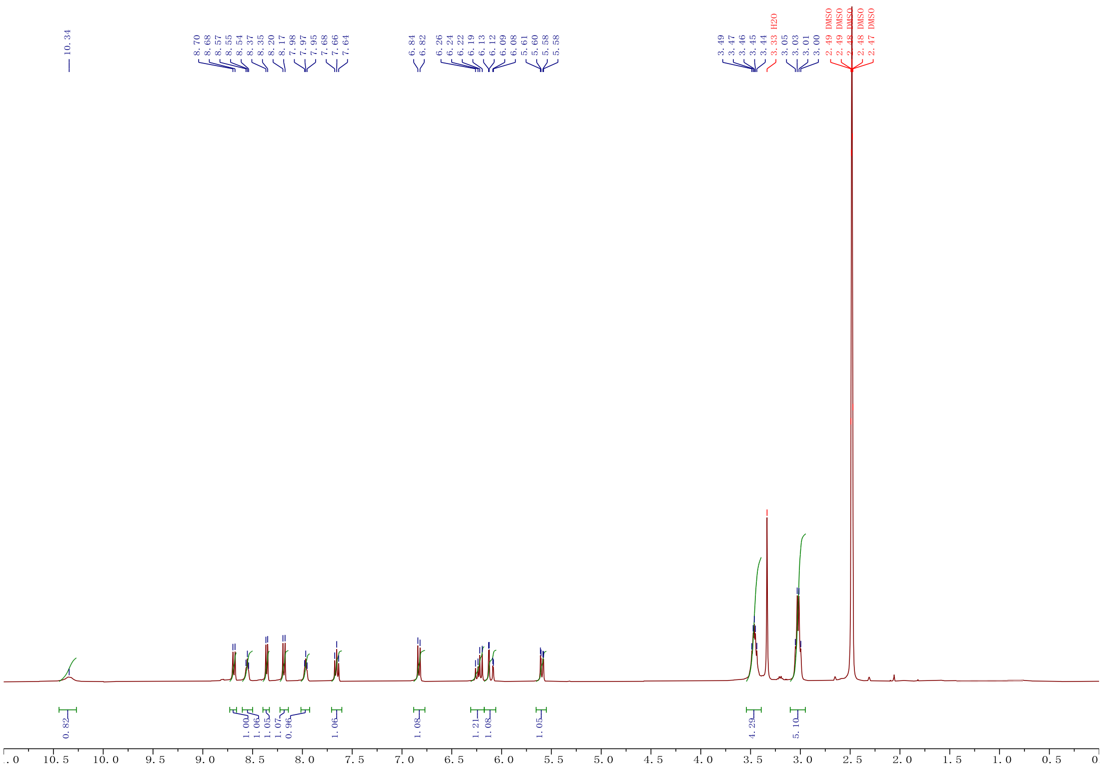

^{1}H NMR spectrum of compound D2

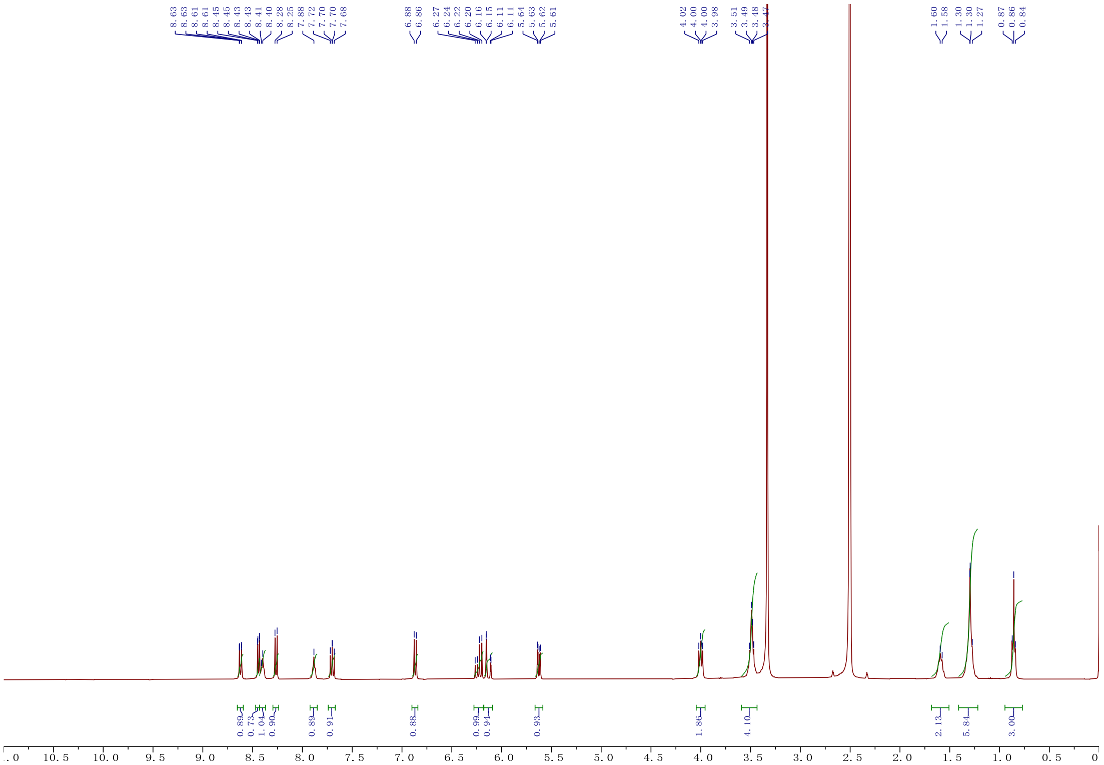

^{1}H NMR spectrum of compound D3

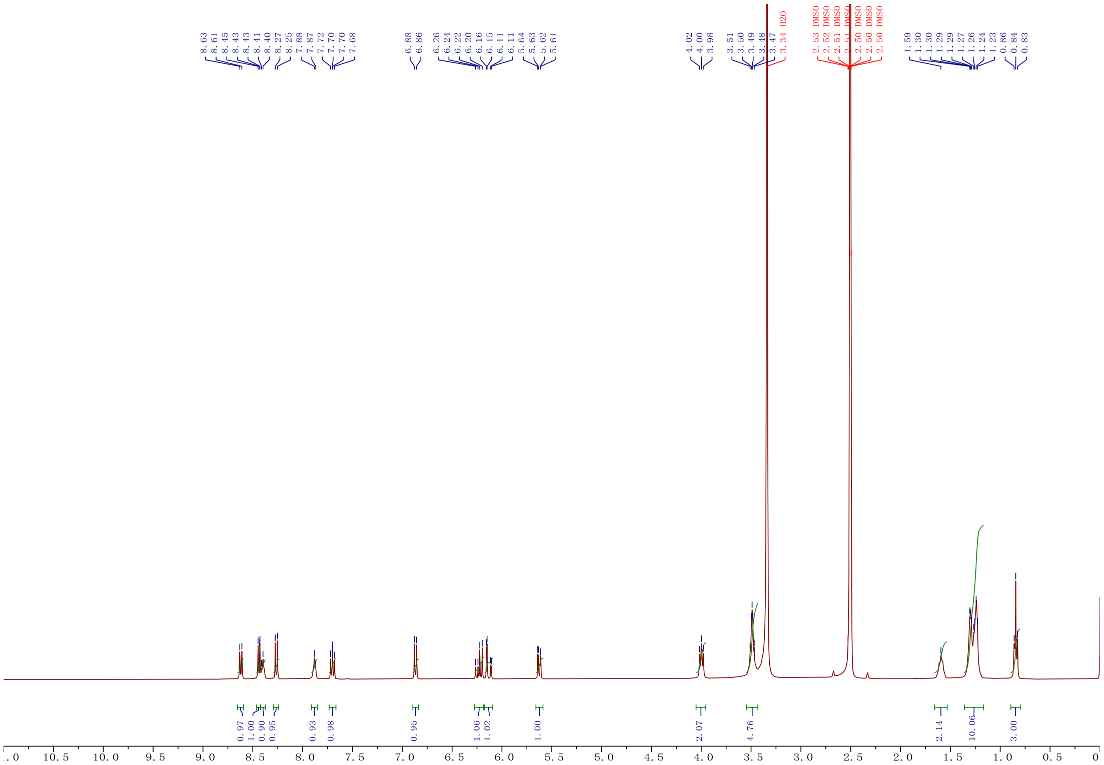

^{1}H NMR spectrum of compound E1

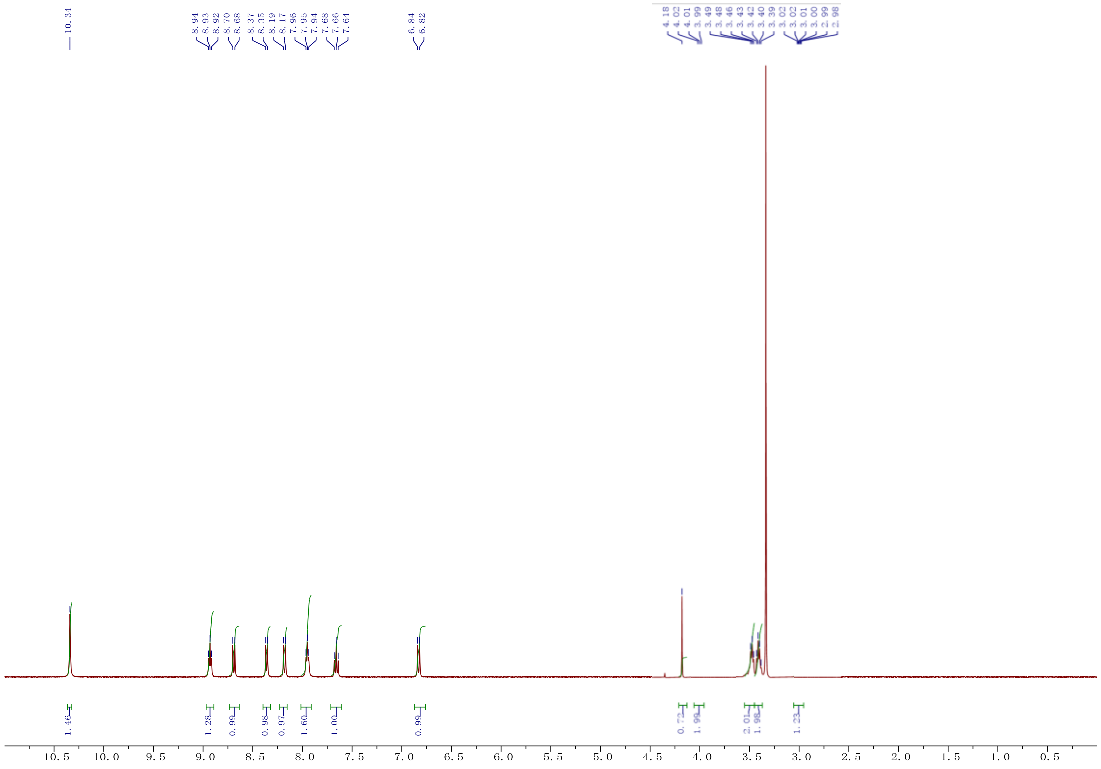

^{1}H NMR spectrum of compound E2

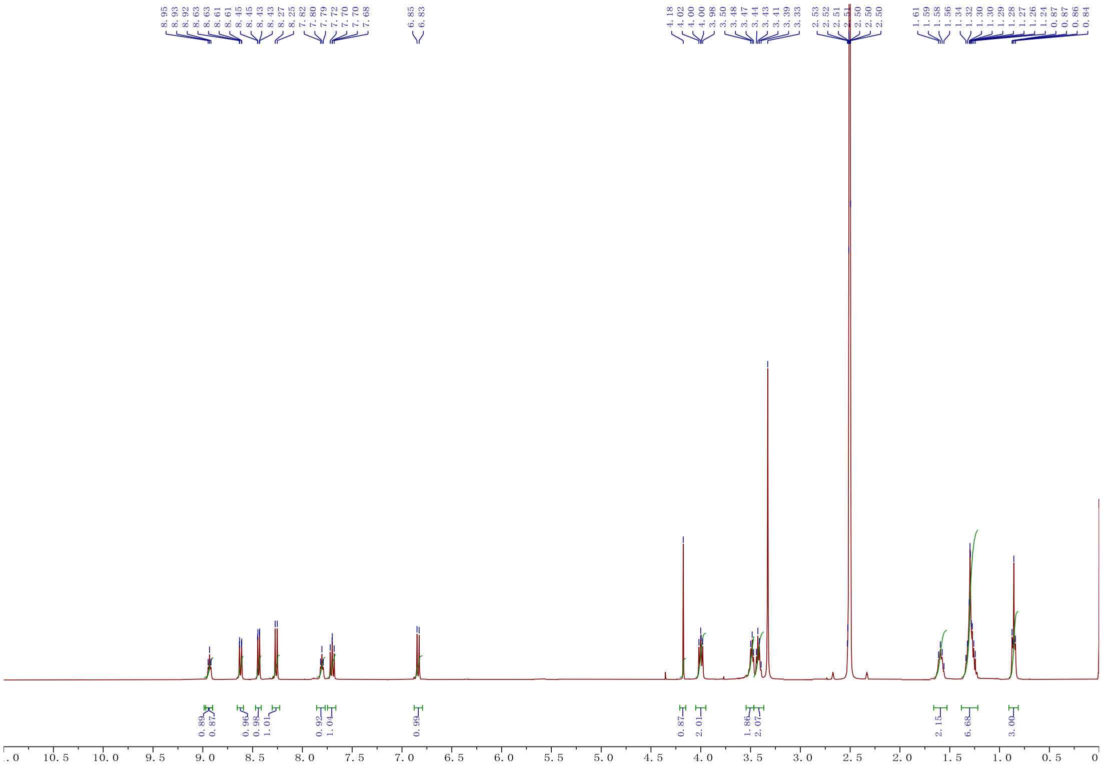

¹H NMR spectrum of compound E3

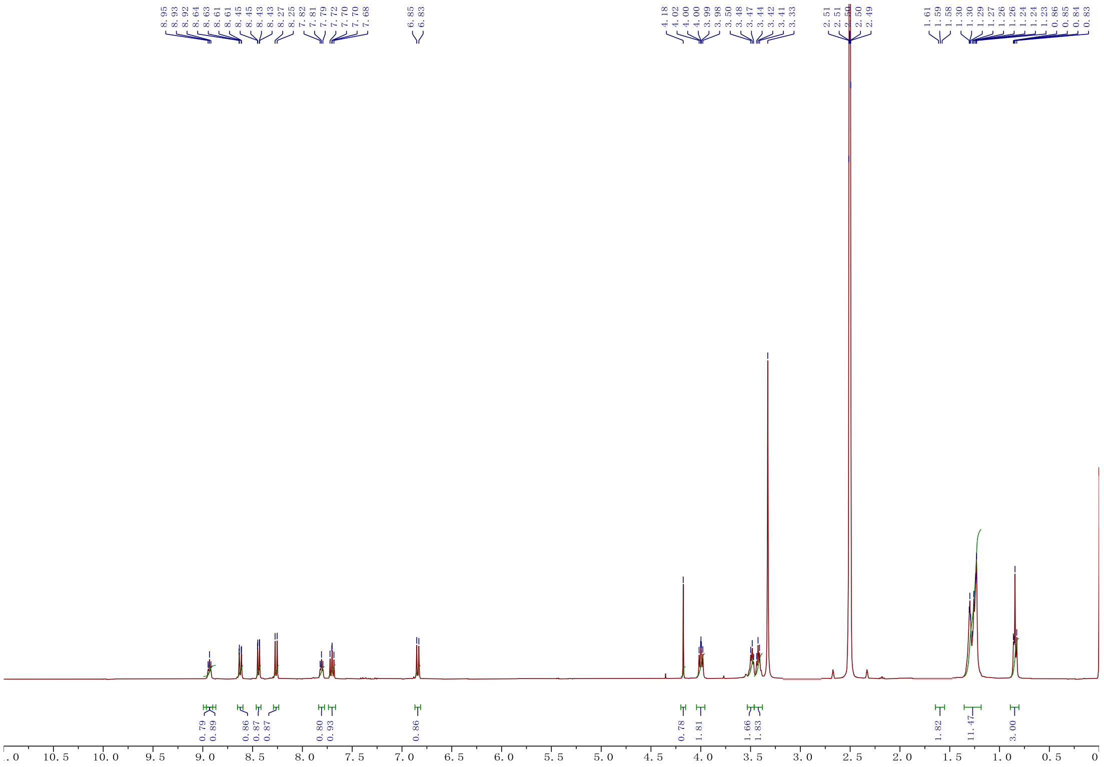

¹H NMR spectrum of compound D3–ibuprofen

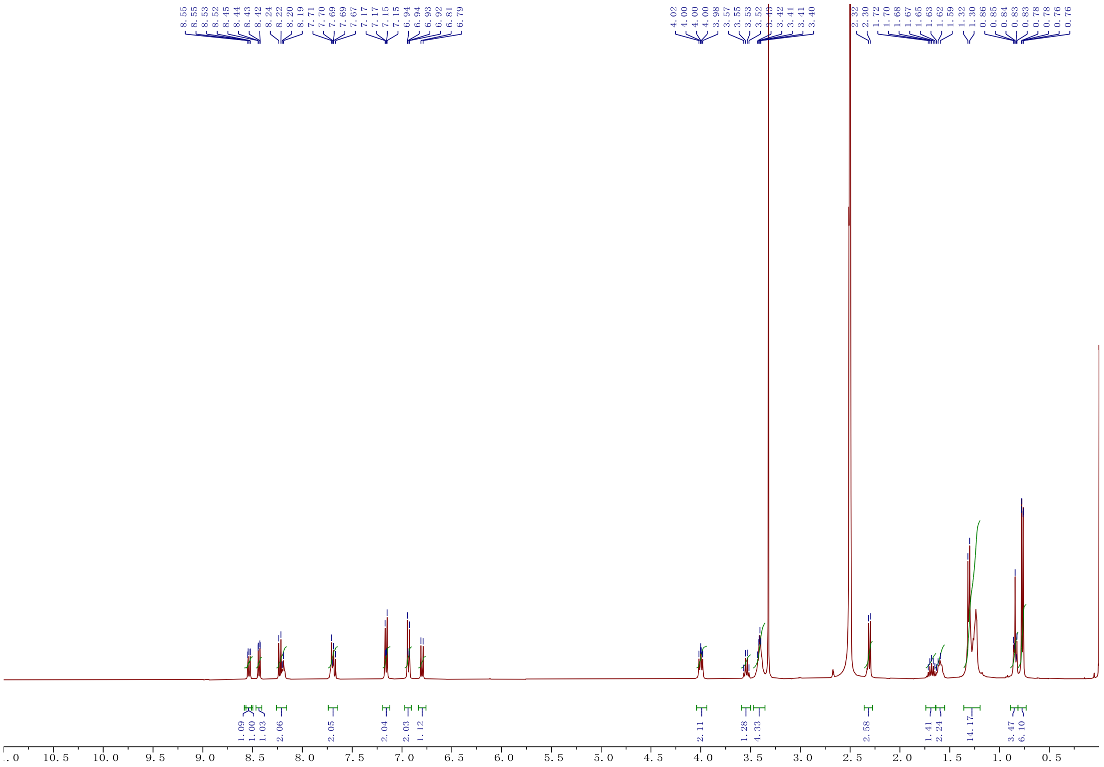

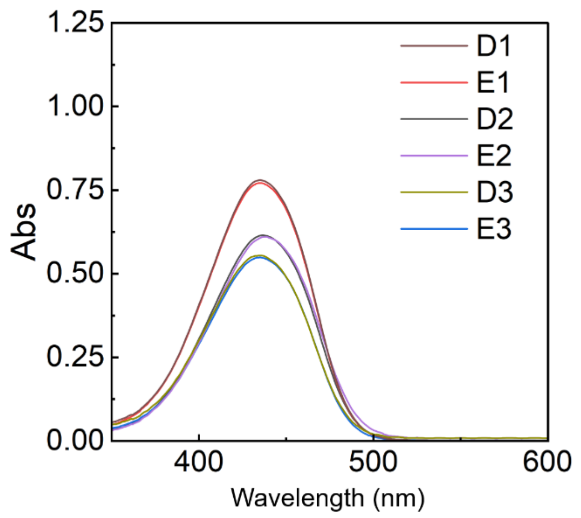

Figure S1. UV–visible absorption spectra of Probe **D1**–**D3** and Probe **E1**–**E3** (5 μM) in PBS containing 1% (v/v) DMSO and 0.2% (v/v) Tween 80. The spectra were recorded over the wavelength range of 350–600 nm.

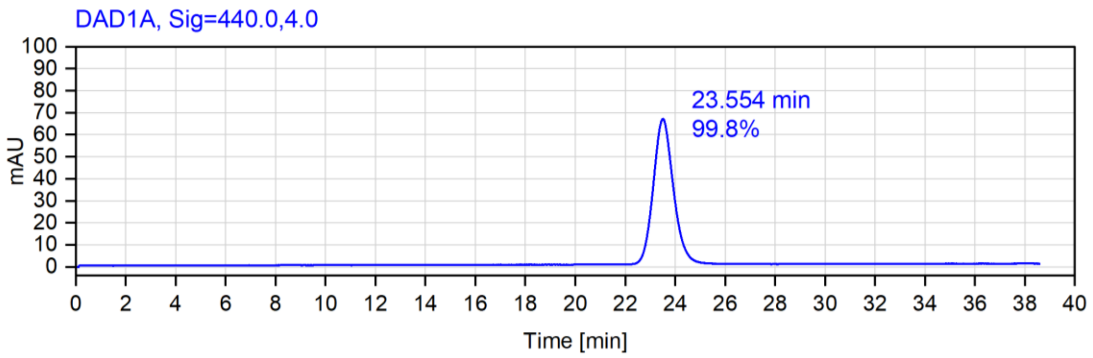

Figure S2. HPLC chromatogram of Probe D3 recorded at a detection wavelength of 440 nm.

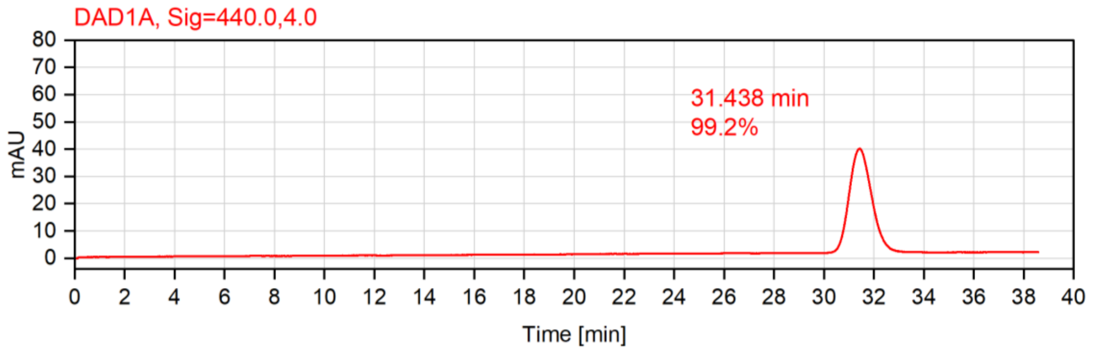

Figure S3. HPLC chromatogram of Probe D3–ibuprofen recorded at a detection wavelength of 440 nm.
